# Incorporation of Multiple β^2^-Hydroxy Acids into a Protein *In Vivo* Using an Orthogonal Aminoacyl-tRNA Synthetase

**DOI:** 10.1021/acscentsci.3c01366

**Published:** 2024-04-23

**Authors:** Noah X. Hamlish, Ara M. Abramyan, Bhavana Shah, Zhongqi Zhang, Alanna Schepartz

**Affiliations:** † Department of Molecular and Cellular Biology, University of California, Berkeley, California 94720, United States; ‡ Schrödinger, Inc., San Diego, California 92121, United States; § Process Development, Attribute Sciences, Amgen Inc., Thousand Oaks, California 91320, United States; ∥ Department of Chemistry, University of California, Berkeley, Calfornia 94720, United States; ⊥ California Institute for Quantitative Biosciences, University of California, Berkeley, California 94720, United States; # Chan Zuckerberg Biohub, San Francisco, California 94158, United States; ¶ ARC Institute, Palo Alto, California 94304, United States

## Abstract

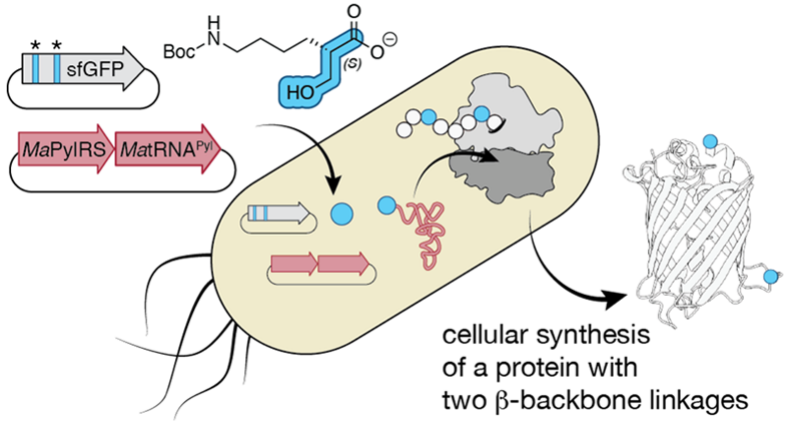

The programmed synthesis of sequence-defined biomaterials whose monomer backbones diverge from those of canonical α-amino acids represents the next frontier in protein and biomaterial evolution. Such next-generation molecules provide otherwise nonexistent opportunities to develop improved biologic therapies, bioremediation tools, and biodegradable plastic-like materials. One monomer family of particular interest for biomaterials includes β-hydroxy acids. Many natural products contain isolated β-hydroxy acid monomers, and polymers of β-hydroxy acids (β-esters) are found in polyhydroxyalkanoate (PHA) polyesters under development as bioplastics and drug encapsulation/delivery systems. Here we report that β^2^-hydroxy acids possessing both (*R*) and (*S*) absolute configuration are substrates for pyrrolysyl-tRNA synthetase (PylRS) enzymes *in vitro* and that (*S*)-β^2^-hydroxy acids are substrates *in cellulo*. Using the orthogonal *Ma*PylRS/*Ma*tRNA^Pyl^ synthetase/tRNA pair, in conjunction with wild-type *E. coli* ribosomes and EF-Tu, we report the cellular synthesis of model proteins containing two (*S*)-β^2^-hydroxy acid residues at internal positions. Metadynamics simulations provide a rationale for the observed preference for the (*S*)-β^2^-hydroxy acid and provide mechanistic insights that inform future engineering efforts. As far as we know, this finding represents the first example of an orthogonal synthetase that acylates tRNA with a β^2^-hydroxy acid substrate and the first example of a protein hetero-oligomer containing multiple expanded-backbone monomers produced *in cellulo*.

## Abstract 2

The aminoacyl-tRNA synthetase PylRS from *M. alvus* acylates tRNA^Pyl^ with β^2^-hydroxy acid substrates and supports their incorporation at multiple sites into a protein *in vivo*.

## Introduction

There is great interest in the synthesis and study of sequence-defined biomaterials whose monomer backbones diverge from those of canonical α-amino acids. Such hetero-oligomers can adopt new and known secondary^1^ and tertiary structures^2−4^ that confer novel functions including enhanced proteolytic resistance^5−7^ and membrane permeability.^8,9^ As a class, sequence-defined biomaterials provide otherwise nonexistent opportunities to expand and evolve protein and biomaterial structure and function and develop improved biologic therapies.^10,11^

**Figure 1:**
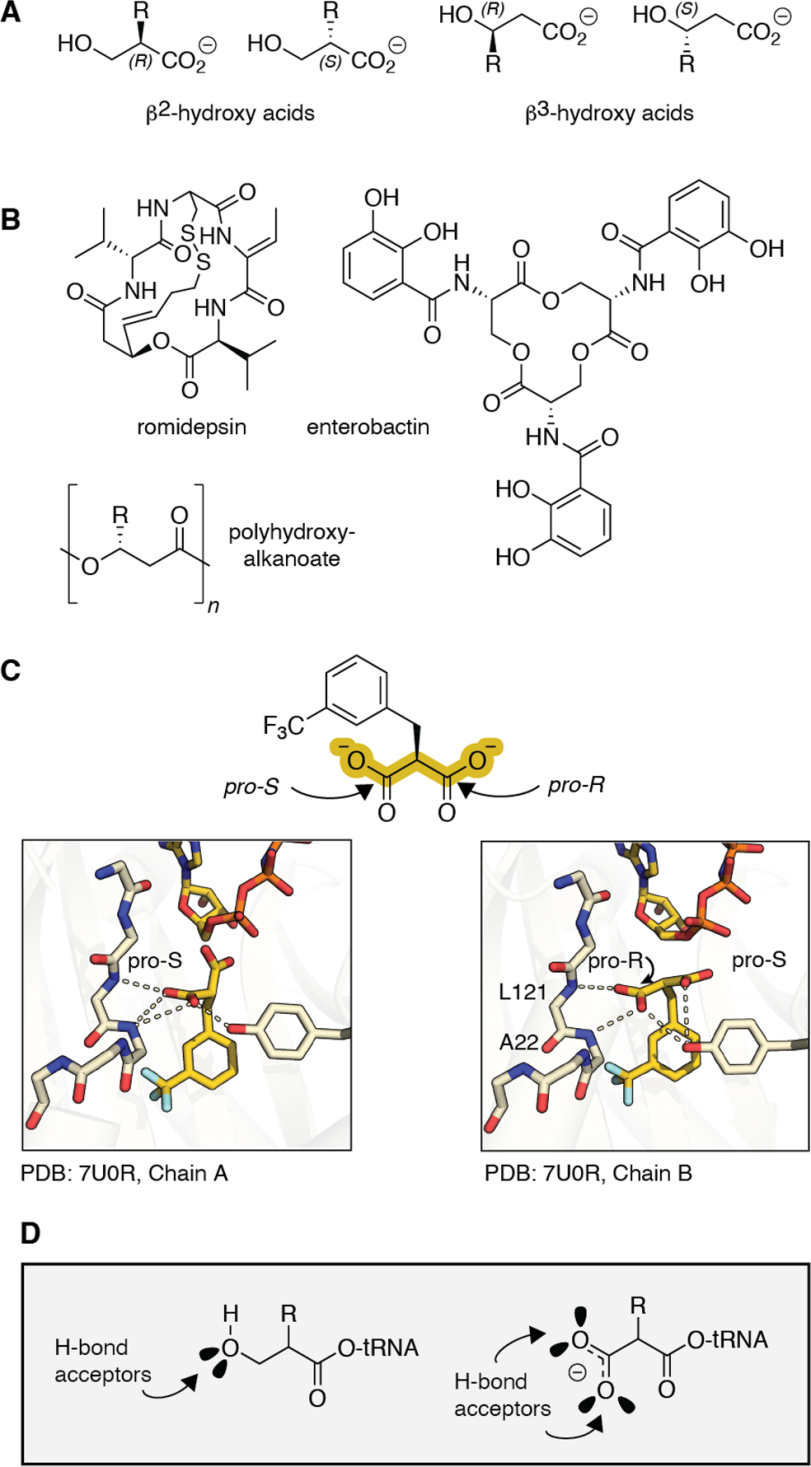
β-Hydroxy acids in natural products and as substrates for pyrrolysyl-tRNA synthetase (PylRS) variants. (A) Structures of β^2^- and β^3^-hydroxy acids and (B) natural products and biomaterials that contain β^2^- or β^3^-hydroxy acid esters. (C) Structure of *Ma*FRSA bound to *m*-CF_3_-2-benzylmalonate (PDB 7U0R) illustrates the absence of a bound active site water and direct H-bonds from L121 and A122 (*Ma* numbering). (D) This work tests the hypothesis that β^2^-hydroxy acids will also act as substrates for PylRS enzymes by virtue of their ability to accept one or two backbone H-bonds.

One monomer family of particular interest for biomaterials consists of β-hydroxy acids (β-HAs) (Figure 1A). β-HAs embody both an expanded backbone and a hydroxy versus amino nucleophile and assemble into polymeric biomaterials known as β-esters. Isolated β-hydroxy acid esters are found in therapeutically relevant natural products (enterobactin),^12^ biosurfactants with environmental applications (surfactin),^13^ and FDA-approved therapeutics (romidepsin) (Figure 1B).^14^ Polymeric β-esters are found naturally in polyhydroxyalkanoate (PHA) polyesters currently in development as bioplastics.^15^

Despite enormous interest in sequence-defined biomaterials containing non-α-amino acid monomers, progress towards their *in vivo* biosynthesis has been exceptionally slow.^16−21^ There exists one report in which a single β^2^-ester has been introduced into a protein using the wild-type *E. coli* ribosome *in vitro*,^17^ but there are no examples in which any β^2^-hydroxy acid has been introduced into a ribosomal product *in vivo*. One challenge limiting the *in vivo* ribosomal synthesis of sequence-defined β-ester biomaterials has been the absence of an orthogonal aminoacyl-tRNA synthetase (aaRS)/tRNA pair that acylates tRNA with a β-hydroxy acid substrate at a level that supports protein biosynthesis.

Previous work has shown that the widely employed and orthogonal pyrrolysyl-tRNA synthetase from *Methanomethylophilus alvus* (*Ma*PylRS),^22,23^ as well as an engineered derivative with two active site Ala substitutions (*Ma*FRSA),^24^ acylate tRNA with a number of non-α-amino acid substrates, including those in which the α-NH_2_ group is replaced with α-H, α-OH, α-SH, α-*N*-methyl, α-*N*-formyl, and α-carboxy substituents.^25−28^ Examination of the structure of *Ma*FRSA bound to one such α-carboxy substrate, *m*-CF_3_-2-benzylmalonate (PDB: 7U0R), provided two insights into how PylRS enzymes might engage expanded backbone monomers such as a β^2^-HA. First, although the structure of PylRS bound to pyrrolysine shows the substrate α-amine coordinated to the homodimeric enzyme in a single conformation via a well-defined active site water molecule,^29^ that water is absent in the structure of *Ma*FRSA bound to *m*-CF_3_-2-benzylmalonate (Figure 1C), presumably to accommodate the expanded size of the α-substituent. In the absence of this bound water, new amide backbone H-bonds from residues L121 and A122 engage the α-carboxy group. Second, in the structure of FRSA bound to *m*-CF_3_-2-benzylmalonate, the two subunits of the dimeric enzyme bind the prochiral *m*-CF_3_-2-benzylmalonate substrate in stereochemically distinct configurations. In one active site, the *pro-S* carboxylate of *m*-CF_3_-2-benzylmalonate engages the backbone amides of L121 and A122; in the other, the substrate rotates, and the *pro-R* carboxylate is coordinated instead (Figure 1C). These results imply that the backbone H-bonds from residues L121 and A122 are highly stabilizing, and PylRS can accommodate substrates retaining the same absolute configuration as natural l-α-amino acids as well as their enantiomers. Some evidence that PylRS accepts certain d-α-amino acid substrates has been reported.^26^

Like the α-carboxy group of a malonic acid, a β^2^-hydroxyl group of tRNA acylated with a β^2^-HA can also accept at least one, and perhaps two, H-bonds from the synthetase amide backbone (Figure 1D). Here we report that β^2^-hydroxy acids possessing both (*R*) and (*S*) absolute configurations are substrates for PylRS enzymes *in vitro*. Further, we report the unexpected finding that only (*S*)-β^2^-hydroxy acids—whose absolute configuration maps onto a d-α-amino acid—are substrates *in cellulo*. Using the orthogonal *Ma*PylRS/*Ma*tRNA^Pyl^ synthetase/tRNA pair and classic *E. coli* expression strains (BL21 and C321.ΔA.exp), we report the cellular synthesis of model proteins containing up to two (*S*)-β^2^-HA residues at internal positions. Metadynamics simulations provide a clear rationale for the observed enantioselective preference for incorporation of an (*S*)-β^2^-hydroxy acid and provide mechanistic insights useful for future translational engineering efforts. As far as we know, this finding represents the first example of an orthogonal synthetase that acylates tRNA with a β^2^-hydroxy acid substrate and the first cellular biosynthesis of a protein hetero-oligomer containing multiple expanded backbone monomers.

## Results

### *Ma*PylRS Acylates tRNA^Pyl^ with a β^2^-Hydroxy Acid Substrate *in Vitro*

**Figure 2:**
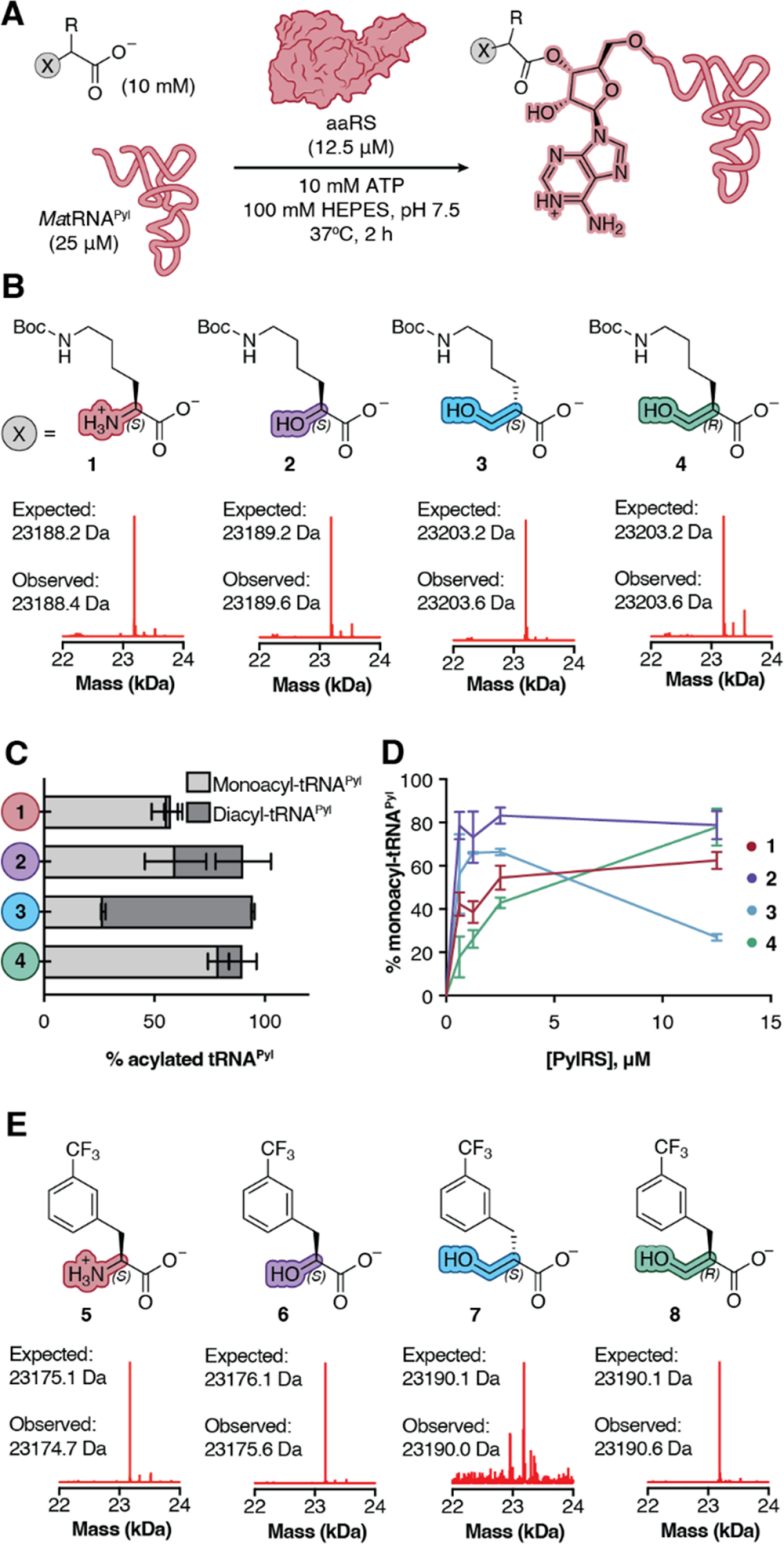
*Ma*PylRS and*Ma*FRSA acylate tRNA^Pyl^ with β^2^-hydroxy acid substrates *in vitro*. (A) Workflow for and (B) deconvoluted mass spectra of *in vitro* tRNA^Pyl^ acylation reactions containing 12.5 μM *Ma*FRSA and supplemented with monomers **1**–**4**. Signal is normalized to the highest signal in the respective traces. Expected masses shown correspond to monoacylated products. (C) Plot illustrating the relative yields of mono- and diacylated tRNA^Pyl^ generated during *in vitro* acylation reactions containing 12.5 μM PylRS and supplemented with 10 mM of monomer **1**, **2**, **3**, or **4**. (D) Plot illustrating yield of monoacylated tRNA^Pyl^ as a function of [*Ma*PylRS]. The analogous plot showing the yield of mono- + diacylated tRNA^Pyl^ as a function of [*Ma*PylRS] is shown in Supporting Information Figure 2. (E) Deconvoluted mass spectra of *in vitro* tRNA^Pyl^ acylation reactions containing 12.5 μM *Ma*FRSA and supplemented with monomers **5**–**8**. Signal is normalized to the highest signal in respective traces. Expected masses shown correspond to monoacylated products. Control tRNA^Pyl^ acylation reactions in which tRNA^Pyl^, enzyme, or substrate is omitted are shown in Supporting Information Figures 1 and 3.

To assess if PylRS-like enzymes would acylate tRNA with a β^2^-hydroxy acid substrate, we performed *in vitro* tRNA acylation reactions using purified enzymes and directly analyzed the products using intact tRNA LC-HRMS (Figure 2A). We began with *Ma*PylRS and compared the yields of acylated tRNA^Pyl^ from reactions containing the known substrates (*S*)-α-NH_2_–N^ε^-Boc-Lysine ((*S*)-α-ΝΗ_2_**1**) and (*S*)-α-OH-N^ε^-Boc-Lysine ((*S*)-α-OH **2**) as well as the enantiopure β^2^-hydroxy acid (β^2^-OH) analogs **3** and **4**. Reactions performed using 12.5 μM *Ma*PylRS, 25 μΜ *Ma*tRNA^Pyl^, and 10 mM **1** or **2** and incubated for 2 h at 37 °C generated the expected monoacylated tRNA products **1**-acyl-tRNA^Pyl^ (23188.4 Da) and **2**-acyl-tRNA^Pyl^ (23189.6 Da) (Figure 2B, Supporting Information Figure 1) in yields of 56% and 59%, respectively (Figure 2C). As observed previously,^27^*Ma*PylRS-promoted acylation of tRNA^Pyl^ with (*S*)-α-OH **2** also generated a diacylated tRNA^Pyl^ product (Figure 2C). Analogous reactions supplemented with (*S*)-β^2^-OH **3** or (*R*)-β^2^-OH **4** also generated the expected diastereomeric monoacylated tRNA products **3**-acyl-tRNA^Pyl^ and **4**-acyl-tRNA^Pyl^ (23203.6 Da) in yields of 27% and 79%, respectively (Figure 2B). Again, under these conditions both substrates also generated detectable levels of diacylated tRNA^Pyl^: 68% for (*S*)-β^2^-OH **3** and 11% for (*R*)-β^2^-OH **4** (Figure 2C). No acylated tRNA^Pyl^ was observed in the absence of enzyme, substrate, or tRNA (Supporting Information Figure 1 and 3). These data imply that β^2^-OH acids **3** and **4** are both substrates for *Ma*PylRS, as anticipated by the FRSA-bound structure of the pro-chiral substrate *m*-CF_3_-2-benzylmalonate (Figure 1C and D).

It has been estimated that the concentration of a single aaRS enzyme expressed from an endogenous promoter in *E. coli* falls in the low μM range.^30^ Thus, these initial reactions, performed at high enzyme concentration (12.5 μM, 50 mol % of tRNA^Pyl^), could mask reactivity differences that are relevant under conditions that better mimic the cellular environment. To more carefully characterize the relative reactivity of monomers **1**–**4**, we evaluated the yield of both mono- and diacylated tRNA^Pyl^ as the concentration of *Ma*PylRS was reduced stepwise from 12.5 μM (50 mol %) to 625 nΜ (2.5 mol %) (Figure 2D). At low PylRS concentrations, the yield of tRNA^Pyl^ monoacylated with (*S*)-β^2^-OH **3** was comparable to that of (*S*)-α-OH **2** and higher than that of (*S*)-α-NH_2_**1** (Figure 2D); at higher enzyme concentrations, the diacylated product predominates. Yields of tRNA^Pyl^ monoacylated with (*R*)-β^2^-OH **4** at low enzyme concentration were slightly lower than yields of tRNA^Pyl^ monoacylated with (*S*)-α-ΝΗ_2_**1**.

### *Ma*FRSA Acylates tRNA^Pyl^ with a β^2^-Hydroxy Acid Substrate *in Vitro*

The PylRS derivative FRSA contains two active site mutations (N166A and V168A, *M. alvus* numbering) that favor substrates with substituted Phe side chains.^24^ One of the best substrates for FRSA is the α-amino acid *m*-CF_3_-Phe **5** (Figure 2E). To determine if *Ma*FRSA would also acylate tRNA with a β^2^-HA, we performed *in vitro* tRNA acylation reactions supplemented with *m*-CF_3_–Phe **5** alongside analogous reactions containing (*S*)-α-OH **6** and the enantiopure β^2^-OH analogs **7** and **8**. Reactions performed with 12.5 μM *Ma*FRSA, 25 μΜ *Ma*tRNA^Pyl^, and 10 mM **5** or **6** cleanly generated the expected monoacylated tRNA products **5**-acyl-tRNA^Pyl^ (23174.7 Da) and **6**-acyl-tRNA^Pyl^ (23175.6 Da) (Figure 2E). Quantification of the monoacyl-tRNA and unacylated tRNA pools indicates yields of 45% and 29% for **5**-acyl-tRNA^Pyl^ and **6**-acyl-tRNA^Pyl^, respectively (Supporting Information Figure 3). Aminoacylation reactions supplemented with 10 mM (*S*)-β^2^-HA monomer **7** yielded only a single low signal peak in the TIC corresponding to the molecular weight of monoacyl-tRNA (23190.0 Da) with a yield of <1% **7**-acyl-tRNA^Pyl^ (Supporting Information Figure 3). By contrast, supplementation of an analogous aminoacylation reaction with (*R*)-β^2^-HA **8** yielded two peaks in the TIC corresponding to monoacylated (23190.6 Da) and diacylated **8**-acyl-tRNA^Pyl^ (23420.1 Da) (Figure 2E) in 28% and 11% yield, respectively. Interestingly, while *Ma*PylRS processes (*S*)-β^2^-OH **3** more efficiently than (*R*)-β^2^-OH **4**, *Ma*FRSA shows the opposite preference, processing (*R*)-β^2^-HA **8** more efficiently than (*S*)-β^2^-HA **7**.

### *Ma*PylRS Supports *in Vivo* Synthesis of a Protein Containing a Single β^2^-HA

**Figure 3:**
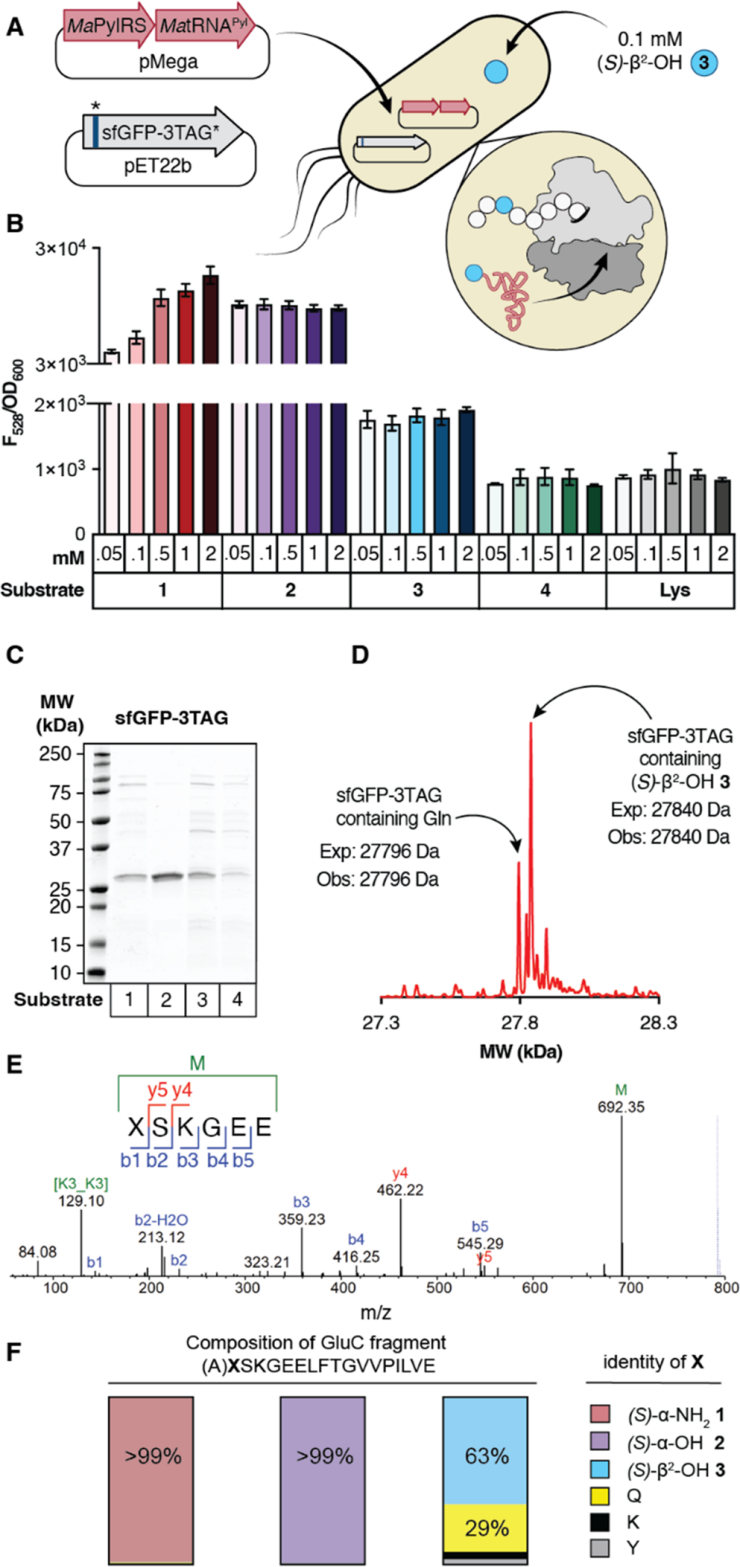
*Ma*PylRS supports *in vivo* synthesis of a protein containing a single β^2^-HA. (A) Workflow for protein expression in C321.ΔΑ.exp *E. coli* transformed with pMega-*Ma*PylRS and pET22b-sfGFP-3TAG. (B) Plot of F_528_/OD_600_ values measured 24 h after induction with 1 mM IPTG as a function of substrate identity and concentration. *n* = 2 biological replicates, 3 technical replicates per biological replicate. (C) SDS-PAGE of sfGFP-3TAG expressed in the presence of **1**–**4**. (D) Deconvoluted mass spectrum of sfGFP-3TAG expressed in the presence of (*S*)-β^2^-OH **3**. (E) MS/MS profile of peptide fragment XSKGEE (where X is (*S*)-β^2^-OH **3**) observed after GluC digestion of sfGFP-3TAG expressed in the presence of (*S*)-β^2^-OH **3**. During collision dissociation, the Boc group of **3** is lost, and subsequent fragmentation generates the commonly observed b and y ions. We note that the analogous peptide fragment corresponding to incorporation of Gln at position 3 (AQSKGEE) is too hydrophilic to be retained on the column and cannot be quantified via LC-MS/MS. (F) Evaluation of the fidelity of incorporation of (*S*)-α-NH_2_**1**, (*S*)-α-OH **2**, or (*S*)-β^2^-OH **3** at position 3 of sfGFP-3TAG using a larger GluC digestion product that could be quantified. Here, **X** denotes position 3, and the preceding A is included when growths included (*S*)-α-NH_2_**1**, Gln, Lys, or Tyr (as the products contain only amide bonds) and not when growths included (*S*)-α-OH **2** or (*S*)-β^2^-OH **3** (as the single ester bond undergoes hydrolysis). Analysis of this longer fragment reveals that more than 99% of the sfGFP-3TAG produced from growths supplemented with (*S*)-α-NH_2_**1** or (*S*)-α-OH **2** contains the requisite monomer at position 3. When the growths are supplemented with (*S*)-β^2^-OH **3**, 63% of the sfGFP-TAG produced contains this monomer at position 3. The remaining material contains Gln (28.6%), Lys (4.4%), or Tyr (2.9%).

We next asked whether the *in vitro* tRNA^Pyl^ acylation efficiencies observed with β^2^-OH substrates would support the incorporation of these monomers into proteins biosynthesized in *E. coli*. Experiments were performed using the recoded *E. coli* strain C321.ΔΑ.exp, which lacks all endogenous TAG codons and release factor 1 (RF1).^31^ Cells were cotransformed with a pMega plasmid^32^ encoding the *Ma*PylRS/*Ma*tRNA^Pyl^ pair (pMega-*Ma*PylRS) as well as a pET22b plasmid encoding sfGFP with an in-frame TAG codon at position 3 (sfGFP-3TAG) (Figure 3A). *Ma*tRNA^Pyl^ naturally decodes TAG codons,^33^ making successful translation of full-length sfGFP dependent on the concentration and activity of acyl-tRNA^Pyl^. Test expressions were performed in the presence of 0.5 to 2 mM **1**–**4**, and both OD_600_ and 528 nm emission (F_528_) were monitored as a function of time (Supporting Information Figure 4). Although the rate of increase in F_528_/OD_600_ was greater for monomers with a natural α-backbone ((*S*)-α-NH_2_**1** and (*S*)-α-OH **2**), by 24 h all growth curves had reached saturation, and hence this time point was used for comparisons of F_528_/OD_600_.

Comparison of F_528_/OD_600_ values after 24 h revealed a clear concentration-dependent increase when cultures were supplemented with (*S*)-α-NH_2_**1** relative to those in which substrate was withheld (ΔAA) or supplemented with Lys, which is not a substrate for *Ma*PylRS (Figure 3B).^34^ Although no concentration dependence of the F_528_/OD_600_ value was observed when cultures were supplemented with (*S*)-α-OH BocK **2**, the F_528_/OD_600_ values observed after 24 h were comparable to those observed in the presence of 0.5 mM (*S*)-α-NH_2_**1**. Cultures supplemented with (*S*)-β^2^-OH **3** also showed an increase in F_528_/OD_600_ relative to those in which the substrate was withheld (ΔAA) or supplemented with Lys. Interestingly, like growths supplemented with (*S*)-α-OH **2**, the F_528_/OD_600_ values observed after 24 h, although low, were independent of the concentration of (*S*)-β^2^-OH **3** over an 80-fold range of concentration. No increases in F_528_/OD_600_ relative to background were observed when cultures were supplemented with (*R*)-β^2^-OH **4**. Identical protein expression assays with C321.ΔΑ.exp cells harboring *Ma*FRSA and supplemented with either enantiomer of β^2^-ΟΗ-*m*-CF_3_-Phe yielded no significant sfGFP expression over a ΔAA control (Supporting Information Figure 5).

Two experiments were performed in an attempt to increase the F_528_/OD_600_ values of cells expressing sfGFP in the presence of (*S*)-β^2^-OH **3**. The growth experiments described above were repeated using either Top10 or BL21 (DE3) *E. coli* in place of C321.ΔΑ.exp. We also examined whether the F_528_/OD_600_ values of growths supplemented with (*S*)-β^2^-OH **3** could be improved via the mutation of *Ma*tRNA^Pyl^. Previous work has emphasized the effect of tRNA identity on the efficiency of noncanonical α-amino acid incorporation into proteins.^35^ A rationally evolved, orthologous tRNA^Pyl^ from the organism *Methanosarcina barkeri* (*Mb*tRNA^Pyl-opt^) bearing mutations at the base of the acceptor and T-stems improved the incorporation of certain noncanonical α-amino acid substrates into proteins expressed in BL21 (DE3) and Top10 *E. coli*,^36^ and mutations at the identical sites in an evolved *Ec*tRNA^Sec^ increased the incorporation of selenocysteine at TAG codons when grown in a derivative strain of C321.ΔA.^37^ Changing neither the expression strain nor the tRNA body improved the observed F_528_/OD_600_ values of growths supplemented with (*S*)-β^2^-OH **3** (Supporting Information Figure 6).

To confirm that (*S*)-β^2^-OH **3** was introduced into sfGFP-3TAG, we isolated protein from a preparative growth of C321.ΔΑ.exp cells transformed with pMega-*Ma*PylRS and pET22b-sfGFP-3TAG and supplemented with 0.1 mM (*S*)-β^2^-OH **3** and characterized its identity using SDS-PAGE (Figure 3C) and LC-HRMS (Figure 3D). SDS-PAGE of cultures supplemented with (*S*)-β^2^-OH **3** produced a major protein product of ∼28 kDa whose mobility was comparable to proteins expressed in the presence of (*S*)-α-NH_2_**1** and (*S*)-α-OH **2** (Figure 3C). The deconvoluted mass spectrum of purified sfGFP-3TAG expressed in the presence of (*S*)-β^2^-OH **3** included a major peak at 27840 Da, corresponding to the expected molecular mass of sfGFP with (*S*)-β^2^-OH **3** at position 3 but lacking residues 1–2 due to ester hydrolysis. A second, smaller peak at 27796 Da was also observed, corresponding to the mass of sfGFP with Gln at position 3; in this case, residue 2 is retained. Thus, although (*S*)-β^2^-OH **3** and (*R*)-β^2^-OH **4** are substrates for PylRS *in vitro*, with activities approaching or exceeding that of (*S*)-α-NH_2_**1**, only (*S*)-β^2^-OH **3** is incorporated into proteins in cells, and with approximately one-tenth the efficiency anticipated on the basis of aaRS activity *in vitro.* Approximately 20–30 mg/L of sfGFP-3TAG could be isolated from C321.ΔΑ.exp cells supplemented with (*S*)-α-NH_2_**1** or (*S*)-α-OH **2**, whereas only 2 mg/L could be isolated from cells supplemented with (*S*)-β^2^-OH **3**. GluC digestion and peptide mapping of sfGFP-3TAG from (*S*)-β^2^-OH **3** supplemented growths provided unambiguous evidence for the presence of the expected β^2^-hydroxy acid at position 3 of sfGFP (Figure 3E) and with reasonable fidelity: roughly 63% of the sfGFP-3TAG produced contains (*S*)-β^2^-OH **3** at position 3 with some contamination with Gln (28.6%), Lys (4.4%), or Tyr (2.9%) (Figure 3F).

**Figure 4:**
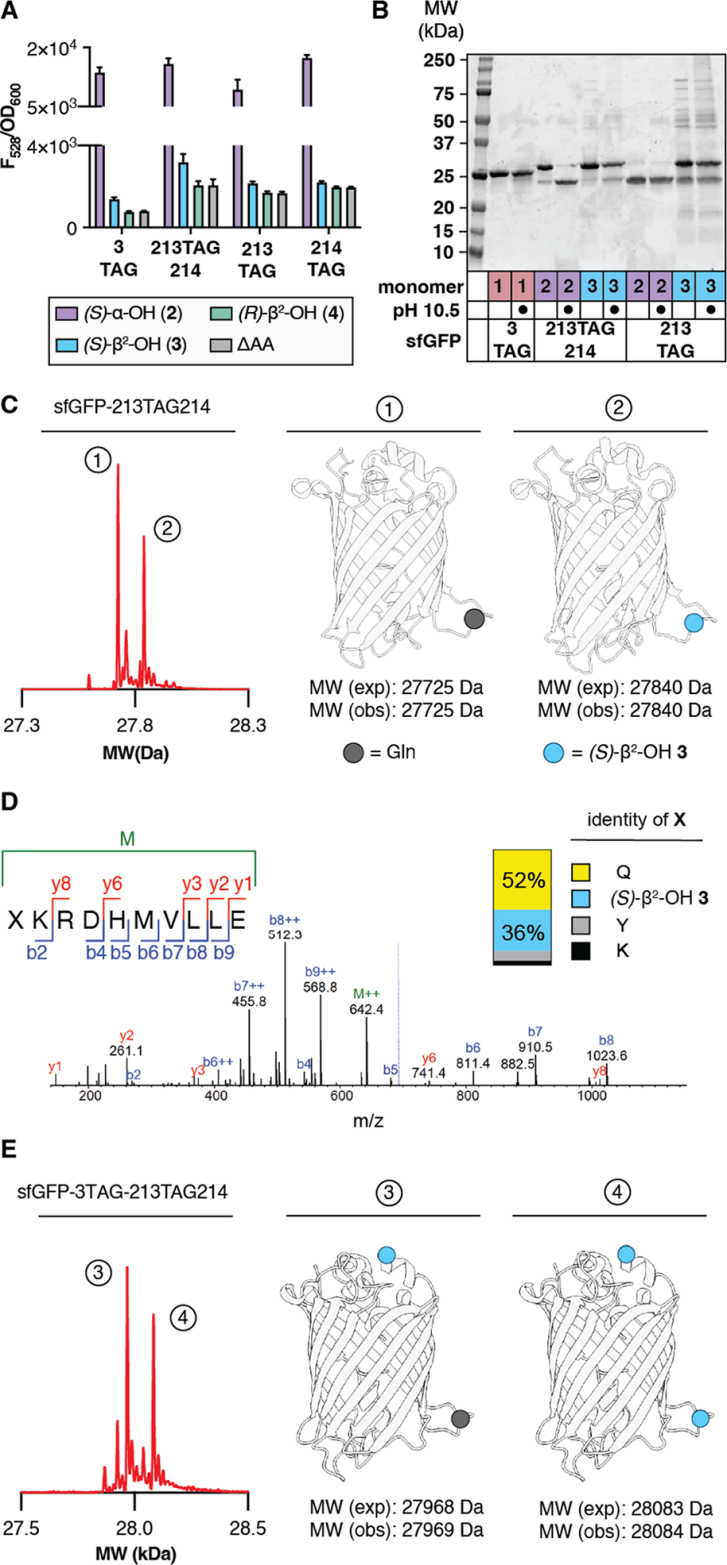
*Ma*PylRS supports the *in vivo* synthesis of sfGFP containing one or two β^2^-hydroxy acid monomers. (A) Plot of F_528_/OD_600_ values of C321.ΔΑ.exp *Ε. coli* transformed with pMega-*Ma*PylRS and the indicated sfGFP expression plasmid measured 24 h after induction with 1 mM IPTG as a function of monomer identity. (B) SDS-PAGE of the indicated isolated sfGFP variants after treatment with CAPS buffer (pH 10.5) or Milli-Q for 2 h at 37 °C. (C) Deconvoluted mass spectrum of sfGFP-213TAG214 expressed in the presence of (*S*)-β^2^-OH **3** reveals two products. One contains a single residue of **3**, and the other contains a single residue of Gln. (D) MS/MS profile for peptide XKRDHMVLLE (where X is (*S*)-β^2^-OH **3**) observed after GluC digestion of sfGFP-213TAG214 expressed in the presence of 0.1 mM (*S*)-β^2^-OH **3**. Inset shows the fidelity of incorporation of (*S*)-β^2^-OH **3** relative to Gln, Tyr, or Lys. (E) Deconvoluted mass spectrum of sfGFP-3TAG-213TAG214 grown in defined media lacking Gln in the presence of 0.1 mM (*S*)-β^2^-OH **3**. Two products are observed, whose masses correspond to (1) sfGFP with the addition of two copies of (*S*)-β^2^-OH **3** and (2) sfGFP with the addition of one copy of (*S*)-β^2^-OH **3** and a single Gln residue. GluC mapping data are found in Supporting Information Figure 9.

To evaluate whether an intact protein containing an intact β^2^-hydroxy acid ester could be isolated, we designed three additional sfGFP expression plasmids. In one, residue E213 was replaced by an amber codon; in a second, residue K214 was replaced by an amber codon; and in the third an amber codon was inserted between E213 and K214. E213 and K214 can function as the N- and C-termini of a split sfGFP variant that assembles from two independent polypeptides.^38^ We reasoned that these sites would be well-suited to accommodate an internal β^2^-hydroxy acid monomer without disrupting either the sfGFP fold or chromophore maturation. C321.ΔΑ.exp cells were cotransformed with pMega-*Ma*PylRS and a pET22b plasmid encoding sfGFP with an in-frame TAG codon at position 213 (pET22b-sfGFP-213TAG), position 214 (pET22b-214TAG), or between positions 213 and 214 (pET22b-213TAG214). Growths were supplemented with 0.1 mM **2**, **3**, or **4** as described previously, and both F_528_ and OD_600_ were monitored as a function of time (Supporting Information Figure 7). Again, although the rate of increase in F_528_ was greater for growths containing (*S*)-α-OH **2** than those containing (*S*)-β^2^-OH **3** or (*R*)-β^2^-OH **4**, by 24 h all growth curves had reached saturation, and this time point was used for comparisons of F_528_/OD_600_. We observed a robust increase in the F_528_/OD_600_ signal of growths expressing sfGFP-213TAG, sfGFP-214TAG, or sfGFP-213TAG214 in the presence of (*S*)-α-OH **2** and a modest increase in F_528_/OD_600_ when cultures expressing sfGFP-213TAG214 were supplemented with (*S*)-β^2^-OH **3** (Figure 4A).

sfGFP variants were isolated from preparative growths of C321.ΔΑ.exp cells transformed with pMega-*Ma*PylRS and either sfGFP-3TAG, sfGFP-213TAG, or sfGFP-213TAG214 and supplemented with (*S*)-α-NH_2_**1**, (*S*)-α-OH **2**, or (*S*)-β^2^-OH **3**. The products were characterized by SDS-PAGE, with and without base treatment (pH 10.5), to confirm the presence of an ester bond (Figure 4B), as well as by LC-HRMS (Figure 4C). SDS-PAGE analysis of sfGFP-213TAG214 isolated from C321.ΔΑ.exp cultures supplemented with (*S*)-α-ΟΗ **2** and without base treatment shows two bands: one that migrates with the MW expected for intact sfGFP (∼28 kDa) and one that migrates with the MW expected for the ester hydrolysis product (∼24 kDa). SDS-PAGE analysis of an analogous sample after base treatment led to virtually complete loss of the 28 kDa band and an increase in the intensity of the 24 kDa band, consistent with the presence of a base-labile ester bond. SDS-PAGE analysis of sfGFP-213TAG214 grown in the presence of (*S*)-β^2^-OH **3** also yielded two bands at ∼24 and 28 kDa prior to base treatment. Base treatment led to a partial loss of the 28 kDa band and an increase in intensity of the 24 kDa band, suggesting that a fraction of the sample contained a base-labile ester linkage. Only a truncated product was isolated from growths programmed with sfGFP-213TAG and supplemented with (*S*)-α-OH **2**; in the case of an analogous growth supplemented with (*S*)-β^2^-OH **3**, both full length and truncated protein was observed, and the ratio was unaffected by base treatment. This observation implies that an ester linkage within sfGFP-213TAG is more hydrolytically labile than an ester linkage within sfGFP-213TAG214.

The successful internal incorporation of (*S*)-β^2^-OH **3** into sfGFP-213TAG214 was confirmed by LC-HRMS. sfGFP isolated from growths programmed with sfGFP-213TAG214 and supplemented with 0.1 mM (*S*)-β^2^-OH **3** contained two sfGFP variants with a yield of approximately 16 mg/L. One variant contained a single residue of **3**, and the other contained a single residue of Gln (Figure 4C). GluC digest followed by peptide mapping to quantitatively assess fidelity indicates that 36% of the sfGFP-213TAG214 produced contains (*S*)-β^2^-OH **3** at position 3. The remaining material contains Gln (52%), Lys (3.6%), or Tyr (8.4%; Figure 4D).

As predicted by gel analysis, sfGFP isolated from growths programmed with sfGFP-213TAG and supplemented with (*S*)-β^2^-OH **3** revealed the presence of Gln at position 213 of sfGFP as well as a pair of protein fragments whose approximate molecular weights (∼4 and ∼23.6 kDa) correspond to those expected if sfGFP was fragmented at position 213. However, the exact mass of the large (23625 Da) fragment was 18 Da less than that predicted on the basis of sequence alone (Supporting Information Figure 8A). It is possible that ester hydrolysis in this case is promoted by the Asn residue at position 212, which could induce ester cleavage via the pathway used by asparagine lyase self-cleaving enzymes, the product of which undergoes dehydration (Supporting Information Figure 8B).^39^

### *Ma*PylRS Supports the *in Vivo* Synthesis of sfGFP Containing Two β^2^-HA Monomers

Having identified that (*S*)-β^2^-OH **3** could be introduced into sfGFP at position 3 as well as between positions 213 and 214, we next asked whether this monomer could be introduced at both positions simultaneously. C321.ΔA.exp cells were transformed with pMega-*Ma*PylRS as well as a plasmid encoding sfGFP with TAG codons at position 3 as well as between E213 and K214 (pET22b-sfGFP-3TAG-213TAG214) and grown in the presence of 0.1 mM (*S*)-β^2^-OH **3** or 0.1 mM (*S*)-α-OH **2**. LC-HRMS analysis of the isolated sfGFP generated in the presence of (*S*)-β^2^-OH **3** confirmed incorporation at both positions, albeit with Gln contamination (Supporting Information Figure 9). However, expression of sfGFP-3TAG-213TAG214 with 0.1 mM (*S*)-β^2^-OH **3** in defined media lacking Gln^27,40^ led to only two products. One contained (*S*)-β^2^-OH **3** at position 3 and glutamine at position 213-214 (27969 Da); the other contained (*S*)-β^2^-OH **3** at both positions (28084 Da) (Figure 4E). GluC digest of sfGFP-3TAG-213TAG214 grown in the presence of 0.1 mM (*S*)-β^2^-OH **3** followed by GluC mapping again unambiguously confirmed the incorporation of (*S*)-β^2^-OH **3** at both positions (Supporting Information Figure 9B).

### Metadynamics Simulation Probe Enantioselectivity of the PTC with Respect to β^2^-OH-Monomers

For over 30 years, the level of noncanonical α-amino acid incorporation at a stop codon has been used as a proxy for aaRS activity *in vivo*.^41^ This proxy fails for the β^2^-backbone monomers studied here. Although *Ma*PylRS acylates tRNA^Pyl^ with both (*S*)-β^2^-OH **3** and (*R*)-β^2^-OH **4** at levels comparable to (*S*)-α-ΝΗ_2_**1** and (*S*)-α-ΟH **2***in vitro*, only (*S*)-β^2^-OH **3** is introduced into protein in cells. We turned to metadynamics to learn more about how (*S*)-α-NH_2_**1**, (*S*)-α-OH **2**, (*S*)-β^2^-OH **3**, and (*R*)-β^2^-OH **4** are accommodated within the ribosomal A-site when loaded on tRNA^Pyl^, as previously reported simulations emphasize the important relationship between nucleophile positioning within the PTC and bond formation.^42^ These metadynamics simulations made use of a reduced ribosome model (RRM) containing fMet-tRNA^fMet^ in the P site and **1-**acyl-tRNA^fMet^, **2-**acyl-tRNA^fMet^, **3-**acyl-tRNA^fMet^, or **4-**acyl-tRNA^fMet^ in the A site. Two 100 ns metadynamics simulations were initiated using two distinct monomer poses (Supporting Information Figure 10), and the results were averaged. One pose aligned the A-site nucleophile with the nucleophilic atom of the A-site Met in the 2.1 Å cryo-EM model used to build the RRM;^42^ this pose placed the α-NH_2,_ α-OH, or β^2^-OH nucleophile as close as possible to the P-site carbonyl. The second pose was generated by rotating the psi (ψ) angle of the A-site nucleophile monomer by 180°; this pose placed the nucleophile farther from the P-site carbonyl.

**Figure 5:**
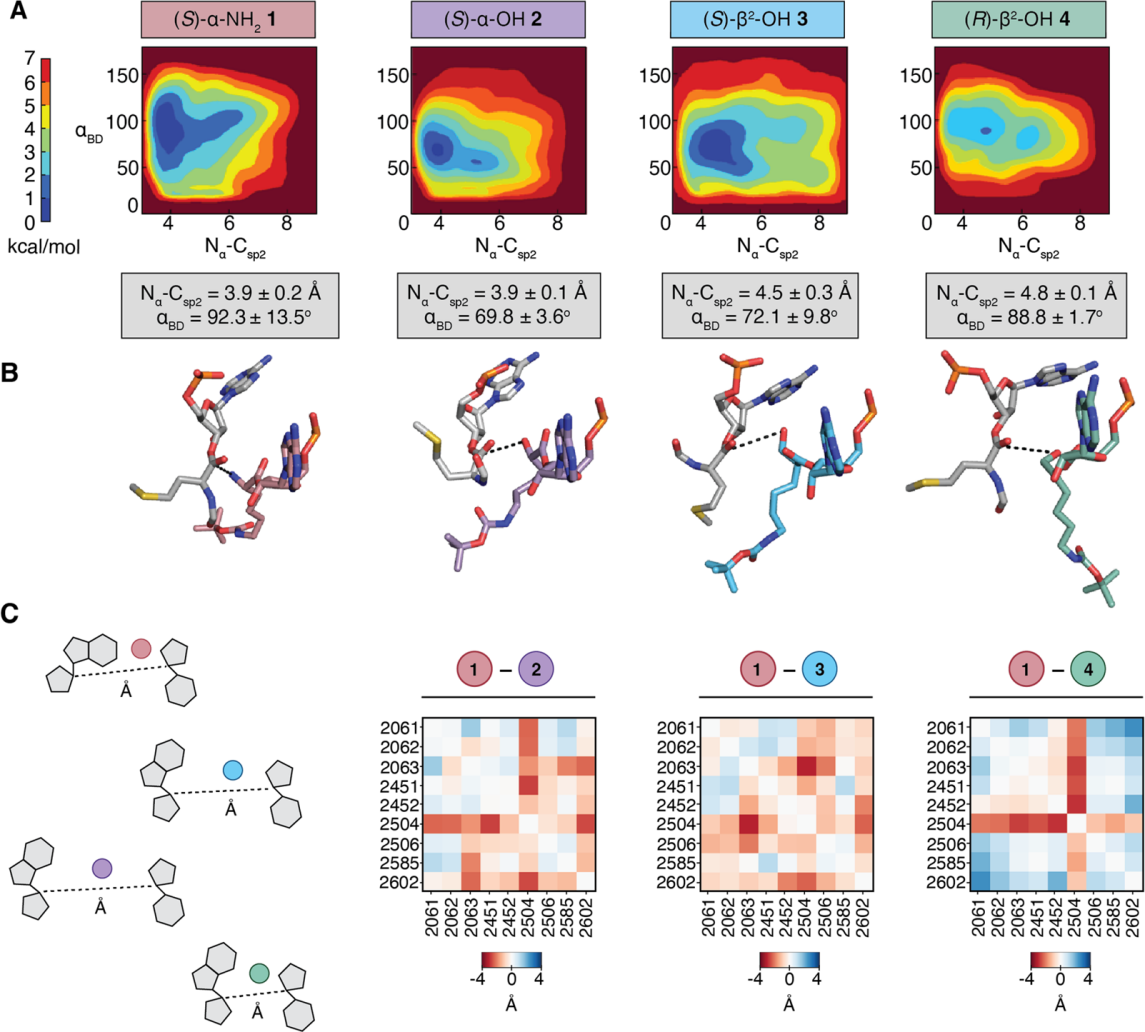
Metadynamics simulations of tRNA acylated with α- and β^2^-hydroxy and amino acids prior to bond formation in a reduced ribosome model (RRM). (A) Free energy surface (FES) plots of 30 Å RRM containing a P-site tRNA^Met^ acylated with fMet and A-site tRNA^Met^ acylated with (*S*)-α-NH_2_**1**, (*S*)-α-OH **2**, (*S*)-β^2^-OH **3**, or (*R*)-β^2^-OH **4**, plotted along the collective variables of Bürgi–Dunitz angle α_BD_ and N_α_–C_sp2_ distance. Each FES shown is the average of two metadynamics runs starting from orientations of the A-site monomers that differ by a 180° rotation about the ψ angle (Supporting Information Figure 10). The color scale represents the free energy in kilocalories per mole (kcal/mol), where the global minima are set at 0 and therefore the various heights of the energy scales are based on the energetics of the fluctuations of the A-site monomers. Average N_α_–C_sp2_ distances and α_BD_ angles for the 0–1 kcal/mol energy minima of each plot are displayed below (gray boxes). (B) Representative conformation and relative geometry of the P-site (gray) and A-site (colored) monomers from the frames at the free energy minimum of each FES plot. Poses were chosen to highlight the N_α_–C_sp2_ distance (black dotted line) and the Bürgi–Dunitz angle α_BD_ at the free energy minimum. The tRNAs to which each monomer is attached as well as the RRM surrounding the two monomers have been omitted for clarity. (C) Pairwise interaction difference analysis (PIA) plots displaying the pairwise-distance differences between the C1′ carbons of nucleotides within 5 Å of each A-site monomer ((*S*)-α-NH_2_**1**, (*S*)-α-OH **2**, (*S*)-β^2^-OH **3**, or (*R*)-β^2^-OH **4**) along the metadynamics trajectories. Heatmaps were constructed by subtracting the C1′ distances within the RRM containing tRNA charged with the indicated monomer from the C1′ distances within the analogous RRM containing (*S*)-α-NH_2_**1**. Darker red indicates greater distances between nucleotides for RRMs containing the indicated monomer ((*S*)-α-OH **2**, (*S*)-β^2^-OH **3**, or (*R*)-β^2^-OH **4**) relative to that containing (*S*)-α-NH_2_**1**, whereas darker blue indicates smaller distances.

Previous results suggest that reactivity within the PTC is related to two distinct parameters: the N_α_–C_sp2_ distance between the A-site nucleophile (N_α_) and the P-site carbonyl electrophile (C_sp2_), and the Bürgi–Dunitz attack angle (α_BD_). Monomers that react readily within the PTC populate a conformational space characterized by a N_α_–C_sp2_ distance of <4 Å and a α_BD_ value between 76 and 115°.^42^ Examination of plots showing α_BD_ as a function of the N_α_–C_sp2_ distance reveals global minima that differentiate highly reactive ((*S*)-α-NH_2_**1**, (*S*)-α-OH **2**), moderately reactive ((*S*)-β^2^-OH **3**), and nonreactive ((*R*)-β^2^-OH **4**) monomers (Figure 5A). The free energy surface for an RRM containing **1-**acyl-tRNA^fMet^ in the A-site is defined by an N_α_–C_sp2_ distance of 3.9 ± 0.2 Å and an α_BD_ of 92.3° ± 13.5° averaged for all final poses within 1 kcal/mol of the global energy minimum (Figure 5B, darkest blue). Both of these values are comparable to those reported for an RRM with Met-tRNA^fMet^ in the A-site (N_α_–C_sp2_ = 3.7 Å and α_BD_ = 76°).^42^ Simulations with **2-**acyl-tRNA^fMet^ in the A-site yielded comparable values (N_α_–C_sp2_ = 3.9 ± 0.1 Å and α_BD_ = 69.8 ± 3.6°), which is fully consistent with the high reactivity of (*S*)-α-OH **2***in vivo*.

The free energy surfaces for an RRM containing **3-**acyl-tRNA^fMet^ or **4-**acyl-tRNA^fMet^ are defined by different global minima values. For the RRM containing **3-**acyl-tRNA^fMet^ in the A-site, we observe low energy poses characterized by N_α_–C_sp2_ distances of 4.5 ± 0.3 Å and α_BD_ values of 72.1° ± 9.8°. The N_α_–C_sp2_ distance for **3-**acyl-tRNA^fMet^ falls outside the N_α_–C_sp2_ distance range for monomers predicted to be highly reactive in the ribosome and suggests that the relatively low incorporation of (*S*)-β^2^-OH **3** is due in part to poor sampling of conformations within the PTC that supports rapid bond formation. The free energy surface of an RRM containing **4-**acyl-tRNA^fMet^ is defined by a similar averaged N_α_–C_sp2_ distance and α_BD_ but with much smaller standard deviations (N_α_–C_sp2_ = 4.8 ± 0.1 Å and α_BD_ = 88.8° ± 1.7°). These differences suggest that **3-**acyl-tRNA^fMet^ can achieve N_α_–C_sp2_ distances that permit modest incorporation of (*S*)-β^2^-OH **3** into a ribosomal product, whereas **4-**acyl-tRNA^fMet^ cannot. Indeed, the energy minima of metadynamics trajectories for monomer **4** produces a minimized structure in which the β-OH of **4-**acyl-tRNA^fMet^ is turned away from the P-site carbonyl electrophile (Figure 5B). This analysis provides one plausible explanation for the observed *in vivo* differences in the reactivity of A-site tRNAs esterified with (*S*)-β^2^-OH **3** and (*R*)-β^2^-OH **4**.

We next examined the overall structure of the PTC during each trajectory to learn more about conformational changes in the ribosome that might facilitate the productive incorporation of (*S*)-β^2^-OH **3** and not (*R*)-β^2^-OH **4**. We first calculated the average distance between the C1′ atoms of all rRNA bases within 5 Å of either acyl-tRNA^fMet^ in the P- or A-site of RRMs containing **1-**acyl-tRNA^fMet^, **2-**acyl-tRNA^fMet^, **3-**acyl-tRNA^fMet^, or **4-**acyl-tRNA^fMet^ (Figure 5C). The matrix of pairwise interactions in each of the four minimized RRMs provides an effective map of how the PTC responds to structurally and stereochemically distinct α-NH_2_, α-OH, and β^2^-OH monomers. To visualize differences between the maps, the pairwise interaction distances calculated from RRMs containing **2-**acyl-tRNA^fMet^, **3-**acyl-tRNA^fMet^, or **4-**acyl-tRNA^fMet^ in the A-site were subtracted from the pairwise interaction distances calculated from the RRM containing **1-**acyl-tRNA^fMet^. The resulting pairwise interaction difference analysis (PIA) plots provide a comprehensive view of how the internal architecture of the PTC varies in a monomer-dependent fashion (Figure 5C).^43,44^ In a PIA plot, distances between rRNA bases that are greater in the presence of **2**, **3-**, or **4**-acyl-tRNA^fMet^ relative to **1**-acyl-tRNA^fMet^ are represented in red, whereas distances that are shorter are represented in blue.

Examination of the PIA plot comparing RRMs containing **1-**acyl-tRNA^fMet^ or **2**-acyl-tRNA^fMet^ shows significant differences at only two rRNA bases (U2504, located within H89, and C2063, part of the highly conserved A2450-C2063 non-Watson–Crick base pair). In both cases, the differences reflect an expansion of the PTC when bound to **2**-acyl-tRNA^fMet^. The PIA plot comparing RRMs containing **1-**acyl-tRNA^fMet^ or **3**-acyl-tRNA^fMet^ is highly similar to the plot comparing the RRMs for **1-**acyl-tRNA^fMet^ and **2**-acyl-tRNA^fMet^ with the largest differences again involving lengthened interactions with A2504 and C2063.

In contrast, the PIA plot comparing the RRMs for **1-**acyl-tRNA^fMet^ and **4-**acyl-tRNA^fMet^ is different. First, it highlights many pairwise interactions that are markedly shorter when **4-**acyl-tRNA^fMet^ occupies the A-site, including those involving U2506, U2585, and A2062. Changes involving U2506 and U2585 are especially notable, as both have been implicated as critical for induced conformational changes required for efficient bond formation.^45^ U2585 is believed to shield the P-site peptidyl-tRNA from hydrolysis in the uninduced state and rotate away in the induced state to expose the ester bond for nucleophilic attack by the A-site monomer.^45^ Indeed, recent cryo-EM structures of *E. coli* ribosomes with aminobenzoic acid monomers in the A-site show U2585 locked in the uninduced conformation, prohibiting access to the P-site peptidyl-tRNA.^46^ Overall, the decreased pairwise distances within the RRM containing **4-**acyl-tRNA^fMet^ may indicate an inability to support the dynamic movements necessary for efficient bond formation. These differences in RRM structure provide a second explanation for the observed *in vivo* enantioselective preference for (*S*)-β^2^-OH **3** over (*R*)-β^2^-OH **4**. More broadly, they emphasize that the complex mechanism of translation, including (but not limited to) EF-Tu- and tRNA-induced conformational changes and essential bound water molecules^46^ must be considered in future ribosome or monomer engineering efforts.

## Discussion

The programmed synthesis of sequence-defined biomaterials whose monomer backbones diverge from canonical α-amino acids is of enormous current interest. Such next-generation molecules provide strategies for improved biologic therapies, tools for bioremediation, and plastic-like materials that biodegrade. But progress toward sequence-defined non-protein materials l has been exceptionally slow.^17−21^ Although most elements of the translational machinery tolerate even wildly divergent α-amino acid side chains,^41^ altered backbones are tolerated predominantly *in vitro*,^47^ at small scale, under nonphysiological conditions, and with efficiencies and fidelities that have not been rigorously evaluated.

Here we report that β^2^-hydroxy acids possessing both (*R*) and (*S*) absolute configuration are excellent substrates for pyrrolysyl-tRNA synthetase (PylRS) enzymes *in vitro* and that certain β^2^-hydroxy acids are also substrates *in cellulo*. One unexpected finding is that the (*S*)-β^2^-hydroxy acid incorporated successfully into protein by the ribosome *in vivo* possesses an absolute configuration that maps onto a d-α-amino acid, not an l-α-amino acid. Although the structure of the PylRS variant FRSA bound to 2-benzylmalonate^27^ provides an explanation for the absence of enantio-preference at the aaRS level, the preference for d-like (*S*)-β^2^-OH **3** in the PTC was a surprise. Metadynamics simulations and recent cryo-EM structures^46^ provide evidence that the preference for tRNA charged with (*S*)-β^2^-OH **3** has less to do with inherent stereochemistry than with differences in the ability to induce conformational changes within the PTC necessary for favorable bond formation. A better understanding of the interactions necessary to facilitate P-site electrophile deshielding could be used to identify d-α-amino acids that are successfully elongated *in vivo*, another long-sought goal.^48^

Overall, the combined biochemical and computational approach reported here provides the first example of orthogonal cellular translation through a β^2^-backbone and the first example of a protein hetero-oligomer containing two β-backbone monomers at predetermined positions. Although further work is necessary to improve yield, understand sequence context, and showcase the benefits embodied by a sequence-defined non-α-peptide backbone, these results represent an important stepping stone toward cell-based synthesis of β^2^-HA/α-AA hybrids with new-to-nature functionalities. They also deepen our understanding of how the WT *E. coli* ribosome accommodates substrates with non-native stereochemistry and backbone configurations (or not) and suggest that translation factor^49,52^ and/or ribosome engineering,^17,50,51^ as well as alternative chemical and biochemical approaches,^19,20^ may be needed to achieve robust levels of expanded backbone linkages within proteins produced in cells.

## Supporting Information Available

Supplementary figures, tables, data, experimental methods, computational methods, and supplementary references (PDF)
