# Correction to “Incorporation of Multiple β^2^‑Hydroxy Acids into a Protein in Vivo Using an Orthogonal Aminoacyl-tRNA Synthetase”

**DOI:** 10.1021/acscentsci.5c00781

**Published:** 2025-06-24

**Authors:** Noah X. Hamlish, Ara M. Abramyan, Bhavana Shah, Zhongqi Zhang, Alanna Schepartz

After this manuscript was published, we became aware that the stereochemistry of the two β^2^-hydroxy acid monomers—*(R)*-β^2^-OH-BocK and *(S)*-β^2^-OH-BocK—had been inadvertently misassigned by the supplier. The correct assignment was inadvertently detected during cryo-EM analysis of ribosomes bound to tRNAs acylated with the two enantiomeric monomers. The molecule we refer to throughout the published paper as *(S)*-β^2^-OH-BocK is in fact *(R)*-β^2^-OH-BocK and *vice versa*. Two additional β^2^-hydroxy acid monomers—*(S)*-β^2^-OH-m-CF_3_-Phe and *(R)*-β^2^-OH-m-CF_3_-Phe (referred to as compounds **7** and **8**, respectively)—were discovered to be enantiopure but without characterization of the absolute configuration of their respective stereocenters.

Although regretful, this finding neither impacts nor alters the two key findings of the published work, namely that (1) *Ma*PylRS acylates MatRNA^Pyl^ with either *(S)*-β^2^-OH-BocK or *(R)*-β^2^-OH-BocK *in vitro*; and (2) in cells, *Ma*PylRS supports the incorporation of two copies of a β^2^-hydroxy acid monomer into protein. The difference is that the monomer introduced in cells is *(R)*-β^2^-OH-BocK not *(S)*-β^2^-OH-BocK.

Although these two key findings are unaffected, the manuscript requires the correction of structures drawn in the Table of Contents graphic, Figures 2, 3, 4, and 5, and Supplementary Figures 1–4, 7–8, and 10–11. It also requires a modest rewording of the paragraphs interpreting the aaRS activity of compounds **7** and **8** as well as the metadynamics simulations. The corrected main text figures and figure legends are provided here along with the reworded paragraphs. Although unrelated, we also noticed that 3 PylRS residues were un- or mislabeled in Figure 1C. A corrected version of that figure is also provided.

**Figure 1:**
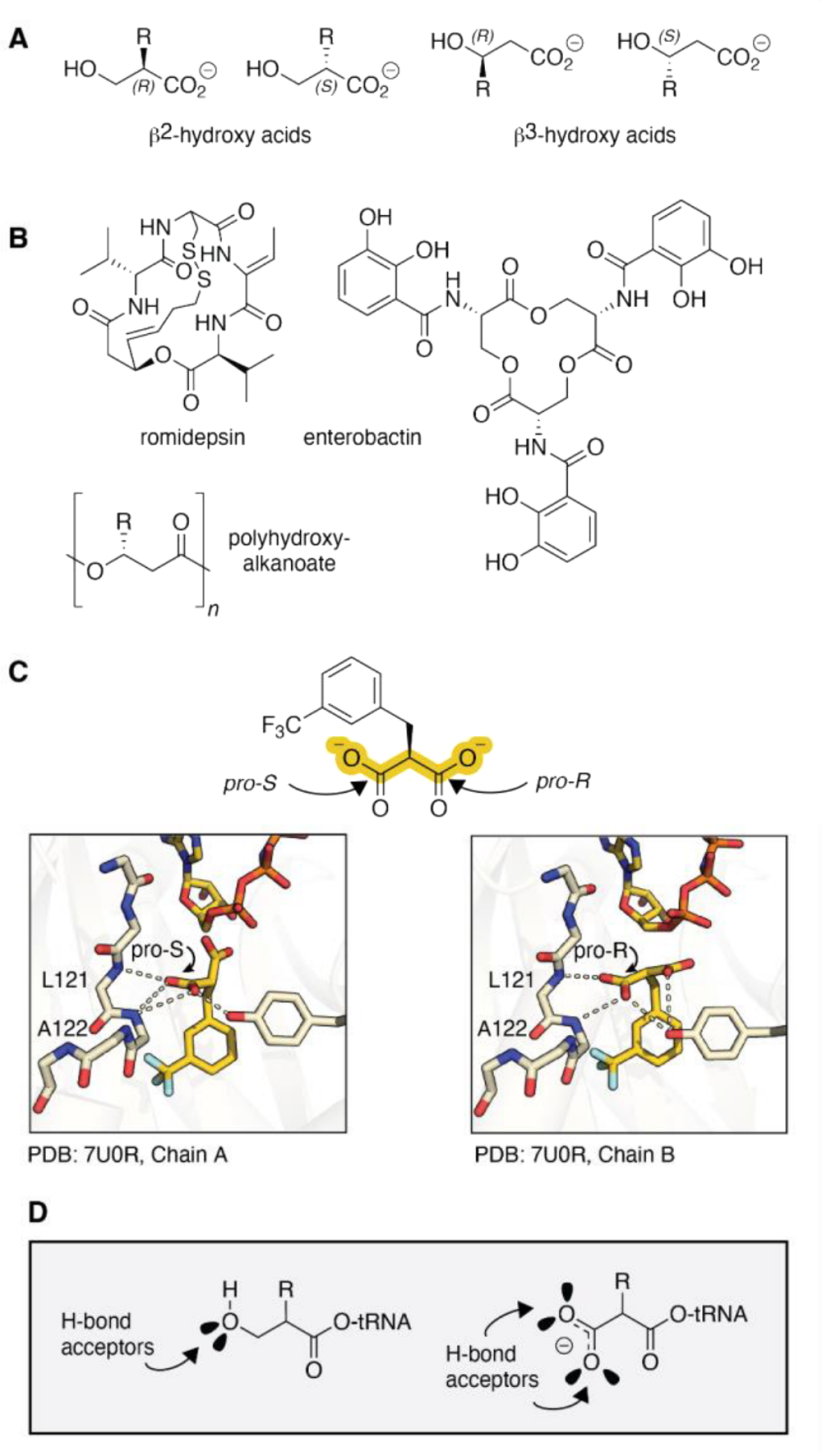
β-Hydroxy acids in natural products and as substrates for pyrrolysyl-tRNA synthetase (PylRS) variants. (A) Structures of β^2^- and β^3^-hydroxy acids and (B) natural products and biomaterials that contain β^2^- or β^3^-hydroxy acid esters. (C) Structure of *Ma*FRSA bound to *m*-CF_3_-2-benzylmalonate (PDB 7U0R) illustrates the absence of a bound active site water and direct H-bonds from L121 and A122 (*Ma* numbering). (D) This work tests the hypothesis that β^2^-hydroxy acids will also act as substrates for PylRS enzymes by virtue of their ability to accept one or two backbone H-bonds.

**Figure 2:**
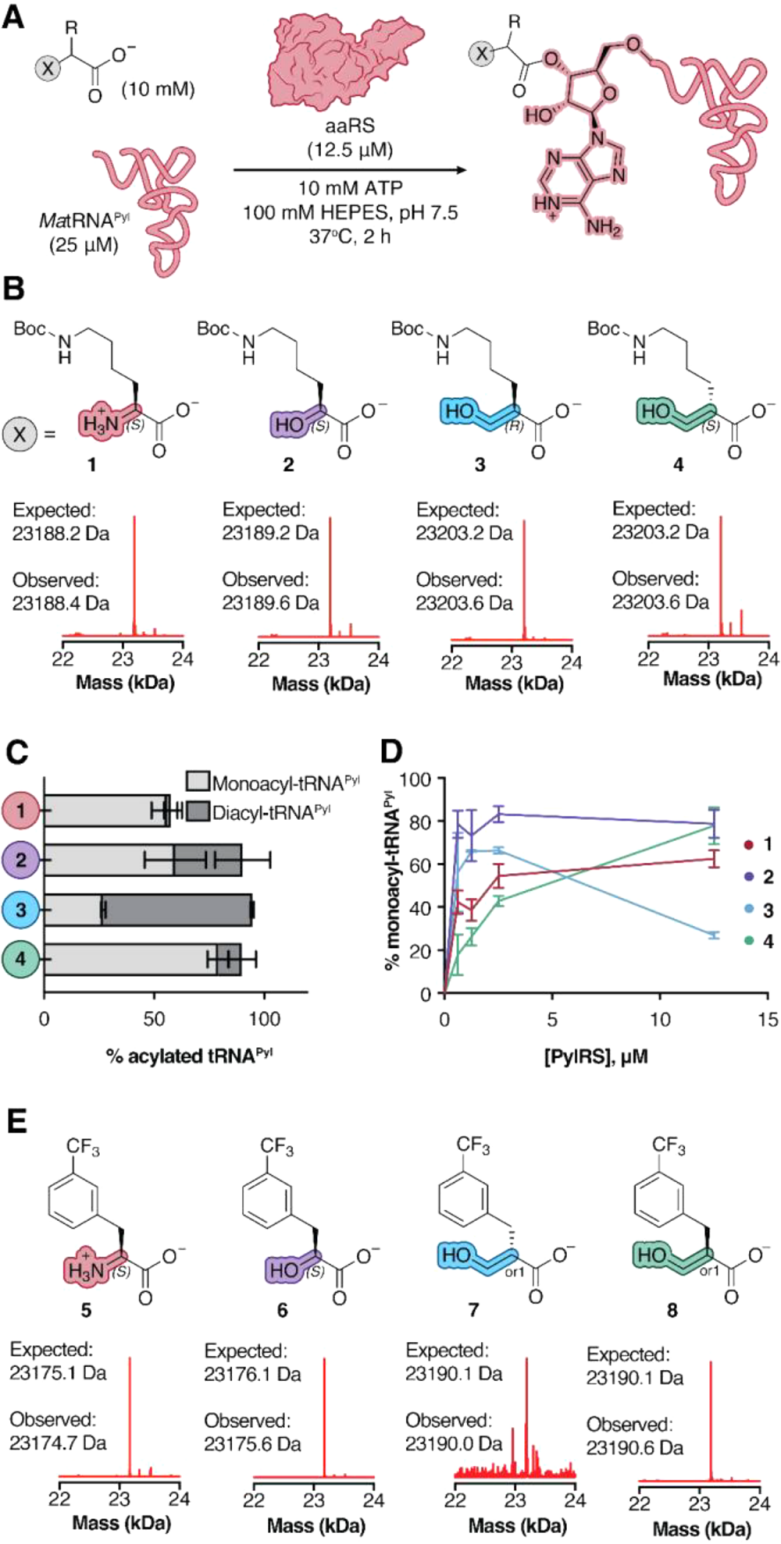
MaPylRS and MaFRSA acylate tRNA^Pyl^ with β^2^-hydroxy acid substrates in vitro. (A) Workflow for and (B) deconvoluted mass spectra of *in vitro* tRNA^Pyl^ acylation reactions containing 12.5 μM MaFRSA and supplemented with monomers **1–4**. Signal is normalized to the highest signal in respective traces. Expected masses shown correspond to monoacylated products. (C) Plot illustrating the relative yields of mono- and diacylated tRNA^Pyl^ generated during in vitro acylation reactions containing 12.5 μM PylRS and supplemented with 10 mM of monomer **1**, **2**, **3**, or **4**. (D) Plot illustrating yield of monoacylated tRNA^Pyl^ as a function of [MaPylRS]. The analogous plot showing the yield of mono- + diacylated tRNA^Pyl^ as a function of [MaPylRS] is shown in Supplementary Figure 2. (E) Deconvoluted mass spectra of *in vitro* tRNA^Pyl^ acylation reactions containing 12.5 μM MaFRSA and supplemented with monomers **5–8**. Signal is normalized to the highest signal in respective traces. Expected masses shown correspond to monoacylated products. Control tRNA^Pyl^ acylation reactions in which tRNA^Pyl^, enzyme, or substrate are omitted are shown in Supplementary Figures 1 and 3.

**Figure 3:**
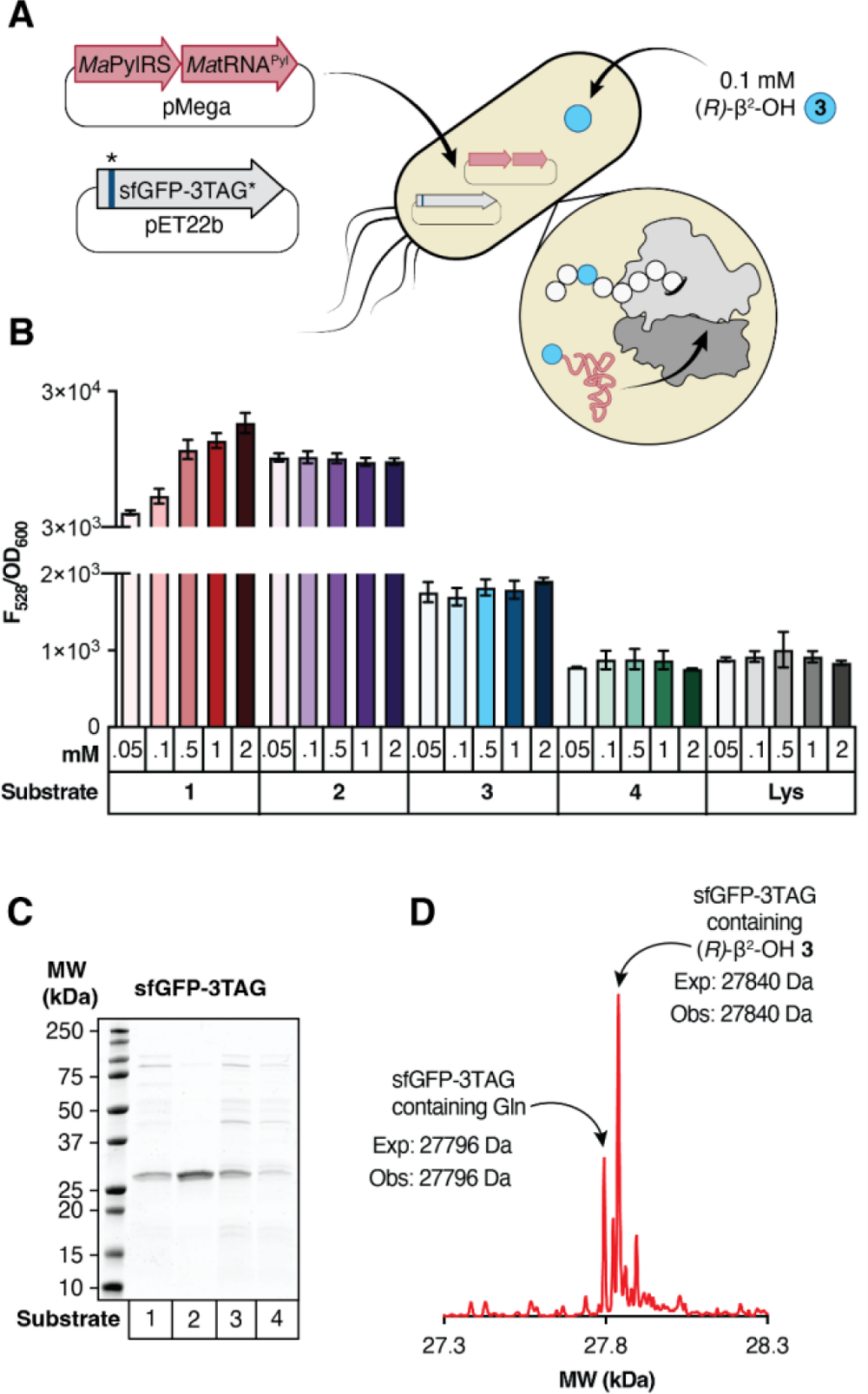
MaPylRS supports *in vivo* synthesis of a protein containing a single β^2^-HA. (A) Workflow for protein expression in C321.ΔΑ.exp *E. coli* transformed with pMega-MaPylRS and pET22b-sfGFP-3TAG. (B) Plot of F_528_/OD_600_ values measured 24 h after induction with 1 mM IPTG as a function of substrate identity and concentration. *n* = 2 biological replicates, 3 technical replicates per biological replicate. (C) SDS-PAGE of sfGFP-3TAG expressed in the presence of **1–4**. (D) Deconvoluted mass spectrum of sfGFP-3TAG expressed in the presence of *(R)*-β^2^-OH **3**. (E) MS/MS profile of the peptide fragment XSKGEE (where X is *(R)*-β^2^-OH **3**) observed after GluC digestion of sfGFP-3TAG expressed in the presence of *(R)*-β^2^-OH **3**. During collision dissociation, the Boc group of **3** is lost and subsequent fragmentation generates the commonly observed b and y ions. We note that the analogous peptide fragment corresponding to incorporation of Gln at position 3 (AQSKGEE) is too hydrophilic to be retained on the column and cannot be quantified via LC-MS/MS. (F) Evaluation of the fidelity of incorporation of *(S)*-α-NH_2_ **1**, *(S)*-α-OH **2**, or *(R)*-β^2^-OH **3** at position 3 of sfGFP-3TAG using a larger GluC digestion product that could be quantified. Here, X denotes position 3 and the preceding A is included when growths included *(S)*-α-NH_2_ **1**, Gln, Lys, or Tyr (as the products contain only amide bonds) and not when growths included *(S)*-α-OH **2** or *(R)*-β^2^-OH **3** (as the single ester bond undergoes hydrolysis). Analysis of this longer fragment reveals that more than 99% of the sfGFP-3TAG produced from growths supplemented with *(S)*-α-NH_2_ **1** or *(S)*-α-OH **2** contains the requisite monomer at position 3. When the growths are supplemented with *(R)*-β^2^-OH **3**, 63% of the sfGFP-TAG produced contains this monomer at position 3. The remaining material contains Gln (28.6%), Lys (4.4%), or Tyr (2.9%).

**Figure 4:**
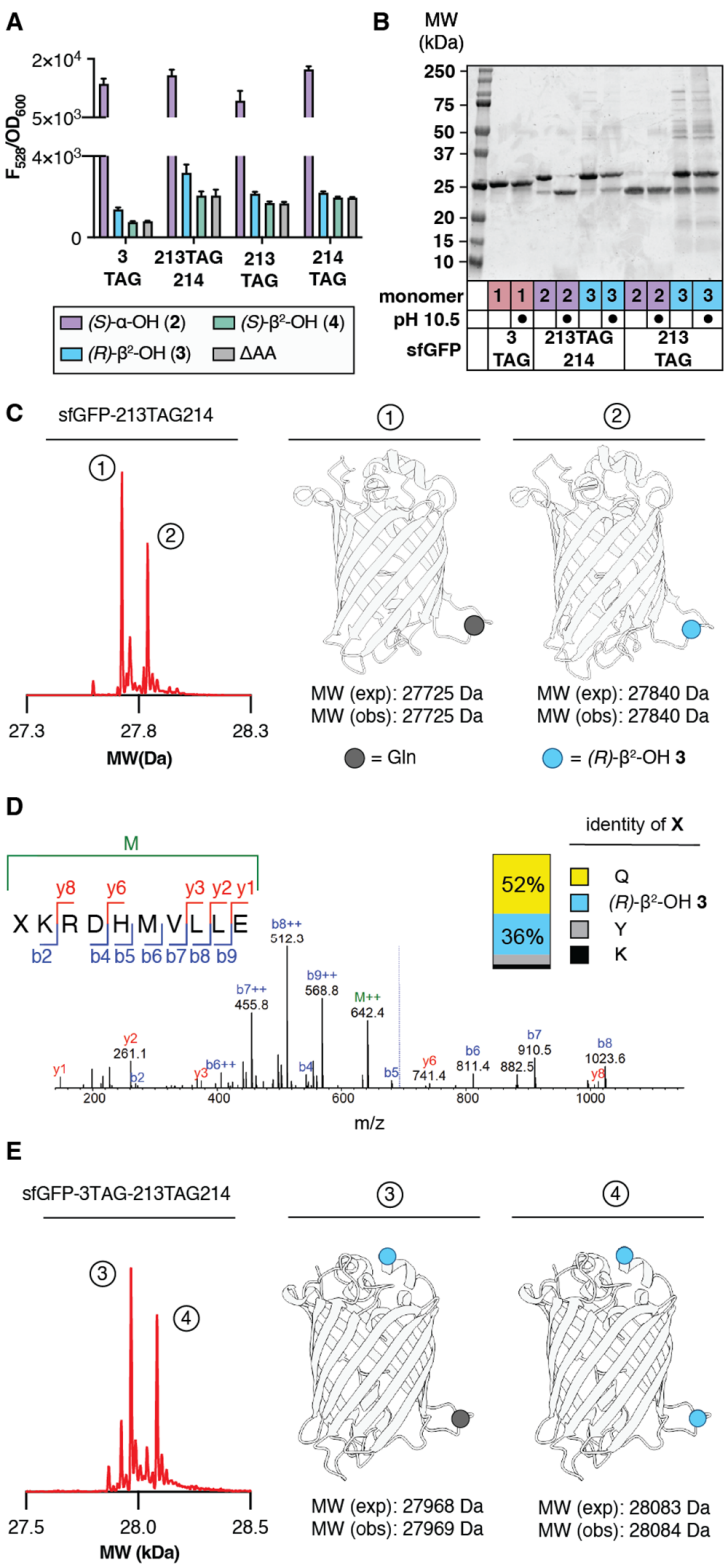
MaPylRS supports the *in vivo* synthesis of sfGFP containing one or two β^2^-hydroxyacid monomers. (A) Plot of F_528_/OD_600_ values of C321.ΔΑ.exp *Ε. coli* transformed with pMega-MaPylRS and the indicated sfGFP expression plasmid measured 24 h after induction with 0.1 mM IPTG as a function of monomer identity. (B) SDS-PAGE of the indicated isolated sfGFP variants after treatment with CAPS buffer (pH 10.5) or Milli-Q for 2 h at 37 °C. (C) Deconvoluted mass spectrum of sfGFP-213TAG214 expressed in the presence of *(R)*-β^2^-OH **3** reveals two products. One contains a single residue of **3**, the other a single residue of Gln. (D) MS/MS profile for peptide XKRDHMVLLE (where X is *(R)*-β^2^-OH **3**) observed after GluC digestion of sfGFP-213TAG214 expressed in the presence of 0.1 mM *(R)*-β^2^-OH **3**. Inset shows the fidelity of incorporation of *(R)*-β^2^-OH **3** relative to Gln, Tyr, or Lys. (E) Deconvoluted mass spectrum of sfGFP-3TAG-213TAG214 grown in defined media lacking Gln and in the presence of 0.1 mM *(R)*-β^2^-OH **3**. Two products are observed whose masses correspond to (1) sfGFP with the addition of two copies of *(R)*-β^2^-OH **3** and (2) sfGFP with the addition of one copy of *(R)*-β^2^-OH **3** and a single Gln residue. GluC mapping data are found in Supplementary Figure 9.

**Figure 5:**
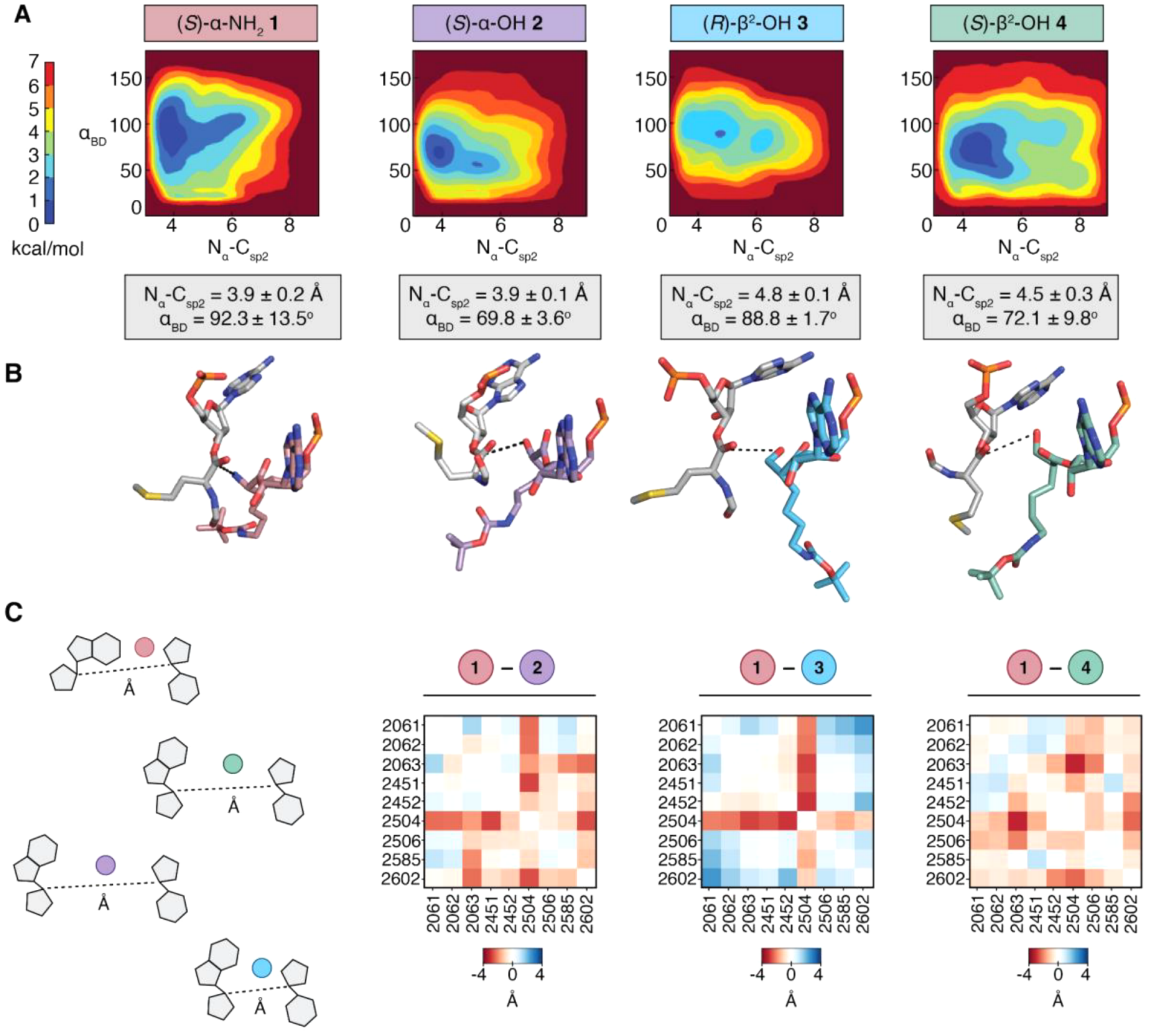
Metadynamics simulations of tRNA acylated with α- and β^2^-hydroxy and amino acids prior to bond formation in a Reduced Ribosome Model (RRM). (A) Free Energy Surface (FES) plots of 30 Å RRM containing a P-site tRNA^Met^ acylated with fMet and A-site tRNA^Met^ acylated with *(S)*-α-NH_2_ **1**, *(S)*-α–OH **2**, *(R)*-β^2^-OH **3**, or *(S)*-β^2^-OH **4**, plotted along the collective variables of Bürgi–Dunitz angle α_BD_ and N_α_–C_sp2_ distance. Each FES shown is the average of two metadynamics runs starting from orientations of the A-site monomers that differ by a 180° rotation about the ψ angle (Supplementary Figure 10). The color scale represents the free energy in kilocalories per mole (kcal/mol), where the global minima are set at 0 and therefore the various heights of the energy scales are based on the energetics of the fluctuations of the A-site monomers. Average N_α_-C_sp2_ distances and α_BD_ angles for the 0–1 kcal/mol energy minima of each plot are displayed below (gray boxes) (B) A representative conformation and relative geometry of the P-site (gray) and A-site (colored) monomers from the frames at the free energy minimum of each FES plot. Poses were chosen to highlight the N_α_–C_sp2_ distance (black dotted line) and the Bürgi–Dunitz angle α_BD_ at the free energy minimum. The tRNAs to which each monomer is attached as well as the RRM surrounding the two monomers have been omitted for clarity. (C) Pairwise Interaction difference Analysis (PIA) plots displaying the pairwise-distance differences between the C1^’^ carbons of nucleotides within 5 Å of each A-site monomer (*(S)*-α-NH_2_ **1**, *(S)*-α–OH **2**, *(R)*-β^2^-OH **3**, or *(S)*-β^2^-OH **4**) along the metadynamics trajectories. Heatmaps were constructed by subtracting the C1^’^ distances within the RRM containing tRNA charged with the indicated monomer from the C1^’^ distances within the analogous RRM containing (S)-α-NH_2_ **1**. Darker red indicates greater distances between nucleotides for RRMs containing the indicated monomer ((S)-α–OH **2**, (R)-β^2^-OH **3**, or (S)-β^2^-OH **4**) relative to that containing (S)-α-NH_2_ **1**, whereas darker blue indicates smaller distances.

## Corrections to Main Figures

Corrections to the table of contents graphic and Figures 1–5 are as follows. Corrected figures appear below.

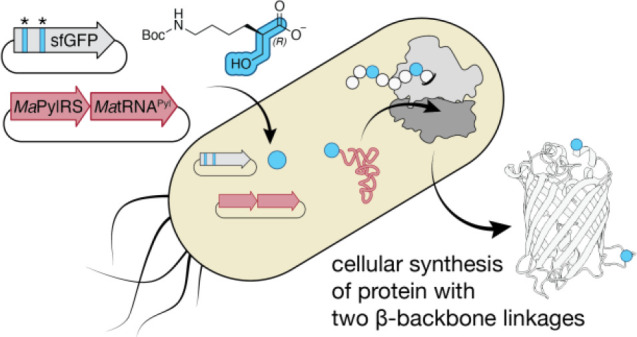



**TOC graphic corrections**: The structure of the monomer shown was changed to indicate that only the *(R)* stereoisomer is introduced into protein in cells.

**Figure 1 corrections**: Amino acid residues within PylRS in panel C were mislabeled in the original manuscript and have been updated with proper labeling. No corrections to text.

**Figure 2 corrections**: (1) In panel B, the stereochemistry of **3** and **4** has been updated to reflect the correct absolute stereochemistry. (2) In panel E, compounds **7** and **8** have been updated with an “or1” nomenclature as provided by the supplier (Enamine). This nomenclature is used to describe enantiopure compounds of unknown absolute configuration. Please also see the text correction below.

**Figure 3 corrections**: (1) In panel A, labeling for compound **3** has been updated with the correct stereochemistry. (2) In panel D, labeling of the deconvoluted mass spec peak has been updated with correct stereochemistry. (3) In panel F, stereochemistry has been updated for monomer **3**. Text throughout the manuscript has been updated with accurate stereochemistry.

**Figure 4 corrections**: (1) In panel A, labeling for compound **3** has been updated with the correct stereochemistry. (2) In panel C, labeling of the blue ball representing compound **3** has been updated with the correct stereochemistry. (3) In panel D, labeling of monomers in compositional analysis updated with accurate stereochemistry. Text updated to match.

## Corrected Paragraphs

### Correction to Section: “Introduction”, Paragraph 5

Like the α-carboxy group of a malonic acid, a β^2^-hydroxyl group of tRNA acylated with a β^2^-HA can also accept at least one, and perhaps two H-bonds from the synthetase amide backbone (Figure 1D). Here we report that β^2^-hydroxy acids possessing both *(R)* and *(S)* absolute configurations are substrates for PylRS enzymes *in vitro*. Further, we report that only *(R)*-β^2^-hydroxy acids—whose absolute configuration maps onto a l-α-amino acid—are substrates *in cellulo*. Using the orthogonal *Ma*PylRS/*Ma*tRNA^Pyl^ synthetase/tRNA pair and classic *E. coli* expression strains (BL21 and C321.ΔA.exp), we report the cellular synthesis of model proteins containing up to two *(R)*-β^2^-HA residues at internal positions. Metadynamics simulations do not provide a clear rationale for the observed enantioselective preference for the *(R)*-β^2^-hydroxy acid suggesting that this selection may occur at least in part prior to ribosomal translation. As far as we know, this finding represents the first example of an orthogonal synthetase that acylates tRNA with a β^2^-hydroxy acid substrate and the first cellular biosynthesis of a protein hetero-oligomer containing multiple expanded backbone monomers.

### Correction to Section: “*Ma*FRSA Acylates tRNA^Pyl^ with a β^2^-Hydroxy Acid Substrate *in Vitro”*, Paragraph 1

The PylRS derivative FRSA contains two active site mutations (N166A and V168A) that favor substrates with substituted Phe side chains.^24^ One of the best substrates for FRSA is the α-amino acid *m*-CF_3_-Phe **5** (Figure 2E). To determine if *Ma*FRSA would also acylate tRNA with a β^2^-HA, we performed *in vitro* tRNA acylation reactions supplemented with *m*-CF_3_-Phe **5** alongside analogous reactions containing *(S)*-α-OH **6** and the β^2^-OH analogs **7** and **8** (enantiopure monomers whose absolute configurations have not been determined as indicated by the “or1” nomenclature, Figure 2E). Reactions performed with 12.5 μM *Ma*FRSA, 25 μΜ *Ma*tRNA^Pyl^ and 10 mM **5** or **6** cleanly generated the expected monoacylated tRNA products **5**-acyl-tRNA^Pyl^ (23175.6 Da) and **6**-acyl-tRNA^Pyl^ (23176.8 Da) (Figure 2E). Quantification of the monoacyl-tRNA and unacylated tRNA pools indicates yields of 45% and 29% for **5**-acyl-tRNA^Pyl^ and **6**-acyl-tRNA^Pyl^ respectively (Supplementary Figure 3). Aminoacylation reactions supplemented with 10 mM enantiopure monomer **7** yielded only a single low signal peak in the TIC corresponding to the molecular weight of monoacyl-tRNA (23189.5 Da) with a yield of <1% **7**-acyl-tRNA^Pyl^ (Supplementary Figure 3). By contrast, supplementation of an analogous aminoacylation reaction with enantiopure monomer **8** yielded two peaks in the TIC corresponding to monoacylated (23189.5 Da) and diacylated **8**-acyl-tRNA^Pyl^ (23420.1 Da) (Figure 2E) in 28% and 11% yield, respectively. The observation that *Ma*FRSA has an enantiopreference for monomer **8** over monomer **7** is consistent with our observation that the parent enzyme *Ma*PylRS exhibits slightly higher activity for (R)-β^2^-OH **3** over (S)-β^2^-OH **4**.

Reference 24. Wang, Y.-S. ; Fang, X. ; Wallace, A. L. ; Wu, B. ; Liu, W. R. A Rationally Designed Pyrrolysyl-tRNA Synthetase Mutant with a Broad Substrate Spectrum. J. Am. Chem. Soc. 2012, 134, 2950–2953, DOI: 10.1021/ja211972x.22289053 PMC3288562

### Correction to Section: “Metadynamics Simulations Probe Enantioselectivity of the PTC with Respect to β^2^-OH-Monomers”, Paragraph 3

The free energy surfaces for an RRM containing **3-**acyl-tRNA^fMet^ or **4-**acyl-tRNA^fMet^ are defined by different global minima values. For the RRM containing **3-**acyl-tRNA^fMet^ in the A-site, we observe low energy poses characterized by N_α_–C_sp2_ distances of 4.8 ± 0.1 Å and α_BD_ values of 88.8° ± 1.7°. The N_α_–C_sp2_ distance for **3-**acyl-tRNA^fMet^ falls outside the N_α_–C_sp2_ distance range for monomers predicted to be highly reactive in the ribosome and suggests that the relatively low incorporation of *(R)*-β^2^-OH **3** is due in part to poor sampling of conformations within the PTC that support rapid bond formation. The free energy surface of an RRM containing **4-**acyl-tRNA^fMet^ is defined by a similar averaged N_α_–C_sp2_ distance and α_BD_ but with larger standard deviations (N_α_–C_sp2_ = 4.5 ± 0.3 Å and α_BD_ = 72.1° ± 9.8°). Indeed, the energy minima of metadynamics trajectories for monomer **4** produces a minimized structure in which the β–OH of **4-**acyl-tRNA^fMet^ is turned toward the P-site carbonyl electrophile (Figure 5B). These data suggest that there are minimal differences in suitability of tRNAs acylated with *(S)*- and *(R)*-β^2^-OH-BocK as substrates for WT *E. coli* ribosomes, and that a bottleneck prior to bond formation precludes the incorporation of *(S)*-β^2^-OH **4**. In support of this conclusion, recent data indicate that the cellular production of tRNA^Pyl^ acylated with *(S)*-β^2^-OH-BocK is barely detectable, while tRNA^Pyl^ acylated with *(R)*-β^2^-OH-BocK-acyl-tRNA^Pyl^ is highly abundant.^43^

Reference 43. Pressimone, M. A. ; Schissel, C. K. ; Goss, I. H. ; Swenson, C. V. ; Schepartz, A. Monitoring Monomer-Specific Acyl-tRNA Levels in Cells with PARTI. Nucl. Acids Res. 2025, 53, gkaf327, DOI: 10.1093/nar/gkaf327.40335069 PMC12058263

### Correction to Section: “Metadynamics Simulations Probe Enantioselectivity of the PTC with Respect to β^2^-OH-Monomers”, Paragraph 6

In contrast, the PIA plot comparing the RRMs for **1-**acyl-tRNA^fMet^ and **3-**acyl-tRNA^fMet^ is different. First, it highlights many pairwise interactions that are markedly shorter when **3-**acyl-tRNA^fMet^ occupies the A-site, including those involving U2506, U2585, and A2062. Changes involving U2506 and U2585 are especially notable, as both have been implicated as critical for induced conformational changes required for efficient bond formation.^46^ U2585 is believed to shield the P-site peptidyl-tRNA from hydrolysis in the uninduced state and rotate away in the induced state to expose the ester bond for nucleophilic attack by the A-site monomer.^45^ Indeed, recent cryo-EM structures of *E. coli* ribosomes with aminobenzoic acid monomers in the A-site show U2585 locked in the uninduced conformation, prohibiting access to the P-site peptidyl-tRNA^46^ These observations further suggest that *(S)*-β^2^-ΟΗ **4** should be able to function as a suitable monomer for the ribosome. Thus, it is possible that steps prior to translation *per se* contribute to the apparent preference for ribosomal incorporation of *(R)*-β^2^-OH **3** over *(R)*-β^2^-OH **4.** More broadly, they emphasize that the complex mechanism of translation, including (but not limited to) EF-Tu- and tRNA-induced conformational changes and essential bound water molecules^46^ must be considered in future ribosome or monomer engineering efforts.

Reference 45. Martin Schmeing, T. ; Huang, K. S. ; Strobel, S. A. ; Steitz, T. A. An Induced-Fit Mechanism to Promote Peptide Bond Formation and Exclude Hydrolysis of Peptidyl-tRNA. Nature 2005, 438, 520–524, DOI: 10.1038/nature04152.16306996

Reference 46. Majumdar, C. ; Walker, J. A. ; Francis, M. B. ; Schepartz, A. ; Cate, J. H. D. Aminobenzoic Acid Derivatives Obstruct Induced Fit in the Catalytic Center of the Ribosome. ACS Cent. Sci. 2023, 9, 1160, DOI: 10.1021/acscentsci.3c00153.37396857 PMC10311655

### Correction to Section: “Discussion”, Paragraph 2

Here we report that β^2^-hydroxy acids possessing both *(R)* and *(S)* absolute configuration are excellent substrates for pyrrolysyl-tRNA synthetase (PylRS) enzymes *in vitro* and that certain β^2^-hydroxy acids are also substrates *in cellulo*. Unsurprisingly *(R)*-β^2^-OH **3**, the β^2^-OH monomer for which we observe incorporation possesses an absolute configuration that maps onto an l-α-amino acid. One unexpected finding is that *(S)*-β^2^-ΟΗ **4** which possesses an absolute configuration that maps onto a d-α-amino acid is still predicted to occupy a conformation in the ribosomal A-site that is productive for bond formation. A better understanding of the interactions necessary to facilitate P-site electrophile deshielding could be used to identify d-α-amino acids that are successfully elongated *in vivo*, another long-sought goal.^48^

Reference 48. Englander, M. T. ; Avins, J. L. ; Fleisher, R. C. ; Liu, B. ; Effraim, P. R. ; Wang, J. ; Schulten, K. ; Leyh, T. S. ; Gonzalez, R. L. ; Cornish, V. W. The Ribosome Can Discriminate the Chirality of Amino Acids within Its Peptidyl-Transferase Center. Proc. Natl. Acad. Sci. U. S. A. 2015, 112, 6038–6043, DOI: 10.1073/pnas.1424712112.25918365 PMC4434717

## Corrections to Supporting Information

The Supporting Information paragraph and descriptions of changes made to the SI are included at the end of this document. A fully corrected SI is included as a separate document.

We are deeply sorry for the errors in our original manuscript and apologize for any inconvenience that these errors may have caused to others.

## Supplementary Material



## Notes

Supplementary figures, tables, data, experimental methods, computational methods, and supplementary references. **Corrections to SI Figures**: Supplementary Figure 1: Labeling of stereocenters for compounds **3** and **4** has been updated with correct stereochemistry. Supplementary Figure 2: Labeling of compounds **3** and **4** updated with correct stereocenters. Supplementary Figure 3: Labeling of stereocenters for compounds **7** and **8** updated with “or1” nomenclature to represent enantiopure monomers of unknown absolute configuration. Supplementary Figure 4: Structures of compounds used in protein expressions updated with correct stereocenter labels. Supplementary Figure 5: No corrections in figure. Text updated to reflect accurate knowledge of stereochemistry. Supplementary Figure 6: No corrections in figure. Text updated to reflect accurate stereochemistry. Supplementary Figure 7: Stereochemistry for compounds **3** and **4** updated to reflect accurate enantiomers supplemented in protein expressions. Text corrected to match. Supplementary Figure 8: Stereochemistry for compound **3** updated to reflect accurate enantiomer. Text updated to match. Supplementary Figure 9: No corrections in figure. Text updated to reflect accurate stereochemistry. Supplementary Figure 10: Stereochemistry labels in panel B updated to correct configurations. Starting poses are reordered to match monomer coloring throughout text. Supplementary Figure 11: In panel A, the stereochemistry of compound **3** has been updated to reflect the correct enantiomer supplemented in protein expressions. In panel B, the stereochemistry of compound **3** has been updated to reflect the correct enantiomer supplemented in protein expressions. In panel C the stereochemistry of compound **3** has been updated to reflect the correct enantiomer supplemented in protein expressions. Text updated to match (PDF)

